# Evaluation of health impacts of a disability-inclusive graduation programme among ultra-poor people with disabilities in Uganda: secondary analysis of a cluster randomized trial

**DOI:** 10.1016/j.eclinm.2025.103318

**Published:** 2025-07-03

**Authors:** Shanquan Chen, Lena Morgon Banks, Elijah Kipchumba, Calum Davey, Munshi Sulaiman, Hannah Kuper

**Affiliations:** a International Centre for Evidence in Disability, London School of Hygiene & Tropical Medicine, London, WC1E 7HT, United Kingdom; b Trinity College Dublin, Dublin, Ireland; c National Institute of Teaching, London, UK; d Brac Institute of Governance and Development (BIGD), BRAC University, Dhaka, Bangladesh; e Independent Evaluation and Research Cell, BRAC Uganda, Kampala, Uganda

**Keywords:** Disability, Poverty reduction, Healthcare access, Graduation approach, Uganda, Randomized controlled trial

## Abstract

**Background:**

People with disabilities experience significant health inequalities and barriers to healthcare access globally. While poverty alleviation interventions show promise for improving health outcomes, evidence specifically for people with disabilities remains limited. This study evaluated the effectiveness of a disability-inclusive graduation (DIG) programme on health outcomes among ultra-poor people with disabilities in Uganda.

**Methods:**

We conducted a two-arm, parallel cluster-randomized controlled trial in four districts of Northern Uganda. Clusters were randomly assigned to either the DIG intervention (96 clusters) or control group (89 clusters). This analysis focused on households with people with disabilities, as identified by the Washington Group Short Set questions and verified by BRAC programme managers. Households in treatment clusters received up to 18 months DIG intervention between December 2020 and June 2022, combining asset transfers, cash support, skills training, financial inclusion activities, and disability-specific services including rehabilitation and assistive devices. The primary outcome was experience of illness/injury in the past 3 months, assessed at both first follow-up (immediately post-intervention) and second follow-up (about 16 months post-intervention), with secondary outcomes including unmet health needs, mental health status, unmet assistive product needs, and healthcare expenditure. Effects were estimated using linear mixed-effects regression or generalized estimating equations, reporting minimally-adjusted and fully-adjusted mean differences (FAMD) or odds ratios (FAOR) with 95% CIs. The trial was registered with RIDIE (RIDIE-STUDY-ID-626008898983a) and ISRCTN (ISRCTN-78592382).

**Findings:**

At baseline, 691 participants (370 intervention, 321 control) were included. The DIG intervention did not significantly impact overall illness/injury prevalence at either first follow-up (41.18% vs 45.86%, FAOR 0.84, 95% CI 0.58–1.22) or second follow-up (55.65% vs 53.98%, FAOR 1.07, 95% CI 0.74–1.56). However, the intervention demonstrated a progressively strengthening effect on reducing unmet health needs, from marginal improvement immediately post-intervention (FAOR 0.56, 95% CI 0.31–1.02, p = 0.06) to significant reduction at 16 months post-intervention (FAOR 0.4, 95% CI 0.22–0.71, p = 0.002). Notably, the intervention produced temporal potential shifts in disease patterns, with malaria showing contrasting trends between follow-up periods. Sex-differentiated effects emerged by second follow-up, with females in the intervention group experiencing fewer injuries (FAOR for interaction 0.17, 95% CI 0.04–0.74, p = 0.02) but more pain-related conditions compared to males (FAOR for interaction 2.43, 95% CI 1.05–5.59, p = 0.04), though these subgroup findings require replication in future studies. No significant differences were observed in mental health outcomes or health expenditure.

**Interpretation:**

This first randomized evaluation of a disability-inclusive graduation programme demonstrates that while economic empowerment alone may not reduce overall illness prevalence among people with disabilities, it can progressively improve healthcare access over time. The temporal evolution of effects and emerging sex-differentiated impacts highlight the need for sustained support and gender-sensitive approaches in future disability-inclusive poverty reduction programmes, with additional health-specific components to achieve broader improvements in health outcomes.

**Funding:**

PENDA, funded by the UK Foreign, Commonwealth and Development Office.

**Research in context**

**Evidence before this study**

We conducted a systematic search of PubMed, Web of Science, and Scopus databases from inception to August 2024, using search terms including “disability”, “poverty reduction”, “graduation program”, “cash transfer”, and “health outcomes”. We included studies in English examining health impacts of poverty reduction interventions for people with disabilities in low- and middle-income countries. While evidence shows that poverty alleviation programs can improve health outcomes in general populations, research specifically focusing on people with disabilities is limited. Existing studies primarily examined cash transfers, showing improved healthcare utilization in China and Kenya, but comprehensive graduation programs' health impacts for people with disabilities remained unexplored.

**Added value of this study**

This study provides the first randomized controlled trial evidence on health impacts of a disability-inclusive graduation programme among ultra-poor people with disabilities. Our findings reveal that while the intervention did not reduce overall illness prevalence, it progressively improved healthcare access over time, with effects strengthening between immediate post-intervention and 16-month follow-up. The study uniquely captures temporal patterns in health outcomes and identifies important gender differences in program impacts, providing crucial insights for future disability-inclusive interventions.

**Implications of all the available evidence**

Combined with previous evidence, our findings suggest that comprehensive poverty reduction programmes can improve healthcare access for people with disabilities, but achieving broader health improvements requires additional components. Future programs should incorporate enhanced disease prevention strategies, gender-sensitive design elements, and sustained health support while maintaining successful aspects of the graduation approach. Policy makers should consider longer intervention periods and careful monitoring of evolving health risks when designing disability-inclusive poverty reduction initiatives.

## Introduction

Approximately 1.3 billion people live with disabilities globally, experiencing more than double the mortality rate of others, resulting in a 14-year life expectancy gap.^1^, ^2^, ^3^ People with disabilities also experience other health gaps, such as poorer health status, barriers to healthcare access, more expensive yet lower quality healthcare, and poorer treatment outcomes.^1^^,^^4^, ^5^, ^6^, ^7^ These health inequalities stem from multiple factors including impairment-related conditions, widespread discrimination and poverty, inadequate disability-related healthcare provider skills and inaccessible facilities.^8^^,^^9^

Poverty alleviation has been central to disability-inclusive development initiatives due to the strong poverty–disability relationship.^10^^,^^11^ Countries have implemented various interventions to move people with disabilities out of poverty, including cash transfers and disability-inclusive livelihood programmes.^12^ These schemes may also improve health and healthcare access through multiple pathways: enabling coverage of direct and indirect healthcare costs, promoting healthier living conditions and preventative activities, reducing stress-related mental health impacts, and providing linked benefits such as health insurance or transport subsidies.^7^^,^^13^, ^14^, ^15^

While the poverty–health relationship is well-established, evidence that poverty alleviation improves health outcomes in low and middle-income countries (LMICs) remains limited and inconsistent.^16^, ^17^, ^18^, ^19^, ^20^ Moreover, programme characteristics appear to influence effectiveness. Conditional cash transfers, where funds depend on health-related requirements, may particularly benefit health outcomes–showing impacts on HIV prevention, immunization coverage, nutrition, and antenatal care usage.^16^^,^^20^, ^21^, ^22^, ^23^, ^24^ Evidence for unconditional cash transfers is more mixed,^18^ though some reviews found similar impacts to conditional transfers.^19^ Transfer amount adequacy remains a key issue, as benefits often insufficiently cover all costs including healthcare.^24^ There is also a lack of data as intervention studies frequently fail to report health outcomes in LMICs,^23^^,^^25^ especially for people with disabilities, though limited evidence from China and Kenya suggests disability-targeted cash transfers can improve healthcare utilization.^26^, ^27^, ^28^

The Disability-Inclusive Graduation (DIG) program was designed with three primary objectives: to improve sustainable livelihoods among ultra-poor people with disabilities through asset transfers and skills training; to enhance social inclusion through community engagement and empowerment activities; and to improve overall well-being, including health and healthcare access, through emergency health funds and rehabilitation services. This approach responds to the established bidirectional relationship between disability and poverty, addressing both economic challenges and disability-specific barriers simultaneously.^10^ Given this relationship and evidence showing that people with disabilities experience significant health inequalities, we hypothesized that the DIG program would reduce illness prevalence, decrease unmet health needs, improve mental well-being, and optimize healthcare expenditure through its comprehensive approach combining economic empowerment with disability-specific supports.

This study evaluates whether the DIG programme achieves its health-related objectives through a disability-inclusive adaptation of BRAC's established graduation model, which combines asset transfers with complementary support. While previous randomized controlled trials demonstrate these programmes reduce poverty and improve psychological status, few measured health impacts.^29^ Evidence from Bangladesh shows positive effects on child nutrition and adult physical/mental health, but impacts specifically for people with disabilities remain unexamined.^30^^,^^31^ Consistent with the program's objectives and underlying theory of change, this study evaluates the effectiveness of the DIG programme at improving health and healthcare access among people with disabilities in Uganda, examining both immediate and subsequent effects through first and second follow-up assessments. Specifically, we considered whether the scheme was effective at reducing prevalence of physical and infectious diseases and unmet health needs, and improving mental well-being and expenditure on healthcare, and how these effects evolved over time.

## Methods

### Study design and setting

We conducted a two-arm, parallel cluster-randomized controlled trial across four districts in Northern Uganda (Kiryandongo, Gulu, Nwoya, and Oyam), where a predominantly agrarian economy and inadequate healthcare access persists despite ongoing governmental and non-governmental efforts. Clusters were defined as villages containing 10–75 eligible households, with households serving as the observational unit. Cluster randomization was employed due to the village-level components of the DIG programme.

This paper reports on the health-related outcomes from the DIG programme trial. As specified in the published protocol,^32^ the primary outcome of the original trial was per-capita annual household expenditure, with livelihood and social participation as additional outcomes of interest. The current analysis focuses specifically on the health-related secondary outcomes from this trial to evaluate the programme's effects on health status, healthcare access, and related outcomes among people with disabilities. The original sample size calculation was based on detecting differences in per-capita expenditure, but its power to examine the health outcomes was reported in the section of statistical analysis.

### Participants

Eligible households in both intervention and control were identified through a census survey conducted by implementing partners (BRAC, Humanity and Inclusion [HI], and National Union of Women with Disabilities of Uganda [NUWODU]) and verified by BRAC programme managers. Eligibility required meeting at least three of five criteria: (1) having a person with disability, (2) being a female-headed household or dependent on female earnings, (3) having children out of school, (4) poor housing conditions (floor, roof, and wall), and (5) low productive asset endowment (referring to a household's minimal ownership of income-generating assets such as livestock, agricultural equipment, or tools for trade/business that could provide sustainable livelihoods). These criteria were established through stakeholder consultation to ensure appropriate coverage of household poverty variations, and were applied consistently across all clusters regardless of treatment allocation.

In the DIG programme, ultra-poverty is understood as a persistent and structural condition of poverty, consistent with the concept of poverty traps, as opposed to temporary or seasonal poverty caused by short-term shocks. Although the international benchmark for extreme poverty is living on less than USD 1.90 per day, discussions with local stakeholders suggested that this measure alone does not adequately capture the specific circumstances in Uganda. As a result, ultra-poverty was defined using a proxy means test based on the five eligibility criteria outlined earlier.

Disability was assessed through a two-stage process for participants in both intervention and control groups. First, all household members completed the Washington Group Short Set questionnaire,^33^ which has been validated across diverse linguistic and cultural contexts.^34^ Individuals reporting “a lot of difficulty” or “cannot do” in any of six domains (seeing, hearing, walking, cognition, self-care, communication) were screened positive.^33^ Subsequently, a BRAC programme manager verified disability status through follow-up visits for all households meeting eligibility criteria, regardless of treatment allocation.

### Clusters

For intervention delivery, villages were organized into geographical clusters containing 10–75 eligible households. Small villages were merged with adjacent villages based on GPS coordinates, while larger villages were divided using k-means clustering, with each resulting cluster containing minimum 10 eligible households. This spatial clustering approach has precedent in public health research, with similar methods used in the WASH Benefits study.^35^ This approach facilitated implementation of village-level components (Village Savings and Loans Associations [VSLAs] and Village Poverty Reduction Committees [VPRCs]) and was incorporated into sample size calculations through simulations accounting for intraclass correlation, with 185 clusters created from 156 villages.

Each household designated a single ‘project participant’ as the primary recipient of training and enterprise support. This individual, selected through field worker-household discussions prior to randomisation, was responsible for managing the enterprise and programme activities. Women and people with disabilities were prioritized as project participants, even if not household heads, to promote gender equity and disability inclusion. When a person with disability was deemed unable to manage available enterprise options, their primary caregiver was designated instead. Children (below 18 years) were ineligible as project participants.

### Ethics

The study received ethical approval from three institutional review boards: the Mildmay Uganda Research Ethics Committee (Reference: 0604-2020), the London School of Hygiene and Tropical Medicine Research Ethics Committee (References: 22619/RR/21198 and 28134), and the Uganda National Council for Science and Technology (Reference: SS529ES).

Written informed consent was obtained from all participants before each data collection round. Interviewers provided hard copies of participant information sheets and consent forms, reading contents aloud to ensure comprehension. Procedures were adapted for different impairments, including sign language interpretation for participants with profound hearing impairments. Written consent was documented through participant-dated signature or thumbprint, alongside the dated signature of the consent obtainer.

### Randomization and masking

The programme team identified 5300 eligible households within eight BRAC branches. Randomization occurred at cluster level, stratified by BRAC office branch to ensure sufficient programmatic support while minimizing confounding from contextual differences. Within each branch, clusters were ranked by number of project participants with disabilities, with geographical separation and cluster derivation considered to minimize contamination.

**Fig. 1:**
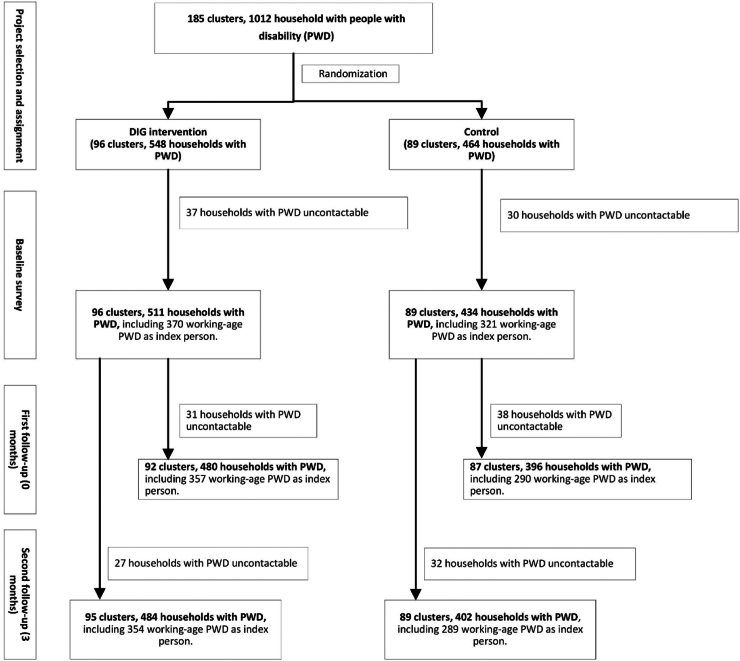
**Trial profile.** DIG, Disability-inclusive graduation programme; PWD, people with disability.

The study used cluster randomization at the village level, not individual or household randomization. Clusters were defined as villages containing 10–75 eligible households, with households serving as the observational unit. While initially aiming for 1:1 allocation within each branch, available funding prioritized serving maximum eligible households (∼2700). Therefore, clusters were randomly assigned to treatment until reaching the desired number per branch, with remaining clusters assigned to control. This resulted in 96 clusters allocated to DIG intervention and 89 clusters to control (Fig. 1). This analysis focuses specifically on the 691 index persons with disabilities (370 intervention, 321 control) from the total eligible population.

Randomization was performed after recruitment by an independent statistician using Stata's random number allocation in October 2020, before baseline data collection. The allocation was stratified by BRAC office branch. While participant and implementer blinding was impossible due to intervention nature, outcome assessors remained masked to allocation. All eligible households within a cluster received the same treatment status (intervention or control) based on their cluster's assignment.

### Procedures

The DIG programme was co-designed over nine months by BRAC, HI, and NUWODU through extensive stakeholder consultation, including people with disabilities, government officials, civil society representatives, and existing Ultra-Poor Graduation (UPG) participants. The design adapted the UPG model using a twin-track approach: providing personalized support for people with disabilities (rehabilitation services, assistive aids, work/home adaptations) while mainstreaming disability inclusion across four graduation pillars.

Intervention households received DIG components between December 2020 and June 2022, with staggered implementation ensuring 18-month delivery periods. Briefly, the intervention included: (1) Livelihoods: Technical training (3-day sessions), asset transfer (e.g., livestock valued at approximately USD 300, typically including 4 female and 1 male goat, or 1 bull/cow, or 2 breeding pigs, or 5 beehives, plus smaller secondary assets), and bi-weekly mentoring on income generation, with assets matched to local opportunities and recipients' capabilities; (2) Social Protection: Six-month cash transfer (USD 18/month), emergency health fund subsidy, rehabilitation services (monthly delivery of occupational therapy, physiotherapy, and psychosocial support for participants respectively), and linkage to existing social entitlements; (3) Financial Inclusion: Financial literacy training (2-day sessions), ongoing coaching, and formation of inclusive Village Savings and Loans Associations (VSLAs); and (4) Social Empowerment: Individual counseling through bi-weekly home visits, life-skills coaching (2-day sessions), and inclusive Village Poverty Reduction Committees (VPRCs).

For participants with disabilities, additional components included assistive devices provision, home and work environment adaptation, and caregiver training in rehabilitation support services. Details of the intervention can be found elsewhere.^32^^,^^36^

Implementation quality was ensured through comprehensive staff training and monitoring throughout both follow-up periods. Ninety project staff underwent initial training in project methodology, disability inclusion, safeguarding, and specific intervention components, with refresher training provided between follow-up periods. Training combined classroom learning with practical field components and external facilitation for specialized topics. The rehabilitation team received specialized training in disability management, mental health, and psychosocial support. Staff competence was assessed through demonstrations and field practice. Regular supervision was conducted by BRAC staff, HI, and NUWODU to maintain intervention fidelity.

Participant adherence was promoted through bi-weekly home visits and regular group meetings, providing tailored guidance and technical support. COVID-19 adaptations included incorporating virtual training while maintaining service continuity. The project operations manual was regularly updated to reflect refined intervention protocols.

We systematically tracked potential serious adverse events and social challenges that might lead to discontinuation, including economic hardships, health issues, and stigma-related barriers. Support measures included economic diversification training, emergency health funds, and community sensitization efforts.

The control group did not receive the DIG intervention but maintained access to existing community programs unrelated to DIG. These existing programs included standard government social services available in the region, such as Uganda's national health services, public education, and community-based agricultural extension services.

Data collection occurred at three time points: baseline (pre-intervention, November 2020), first follow-up (June–July 2022, immediately following the 18-month DIG program), and second follow-up (16 months post-intervention, October–November 2023). At each point, trained data collectors used SurveyCTO, an electronic data collection tool, supervised by BIGD/IERC.

The process comprised two components: Household-level data gathering, including socio-demographic factors, economic status, and disability prevalence; and an in-depth questionnaire on one working-age person with disabilities (‘index person’) per household, focusing on health.

No methodological changes occurred after trial commencement, except for the mental health measurement approach (detailed in Outcomes section).

### Outcomes

The primary outcome was experience of illness/injury, measured by the question “Have you experienced an illness/injury that made you unable to perform normal activities for at least 5 days in the last 3 months?”. This was self-reported and coded as a binary outcome (yes/no). If yes, respondents identified the type of condition from categories including respiratory conditions (TB, pneumonia, fever/flu), gastrointestinal condition (diarrhea, typhoid, vomiting, jaundice), malaria, chronic conditions (fatigue and chronic illness), pain-related conditions (headache, backache, pain), injury, and others.

Secondary outcomes included several measures. First was the experience of unmet health need, defined as a positive response to the primary outcome question (having an illness/injury) but responding ‘no’ to the follow-up question ‘Was treatment sought at a health facility?’. This binary measure captures whether respondents who experienced illness/injury did not seek formal healthcare, regardless of reasons. Mental health status was another key secondary outcome, though measured differently across the study period. At baseline, this was assessed using the Washington Group Extended Set on Functioning (WG-ES) anxiety and depression module, which evaluated the frequency (“never” to “daily”) and severity (“a little” to “a lot”) of feeling worried, nervous, anxious, or depressed. In the first and second follow-up surveys, mental health was measured using the Patient Health Questionnaire (PHQ-9), a validated 9-item questionnaire assessing depressive symptoms over the past two weeks. Scores range from 0 to 27, with higher scores indicating more severe depressive symptoms. In this study, a PHQ-9 total score of ≥10 was considered indicative of potential moderate depression, while a score of ≥15 suggested potential severe depression.

Another secondary outcome was having any unmet assistive products or services needs. This included mobility aids (such as cane, crutches, orthoses, prostheses, rollators, standing frame, therapeutic footwear, tricycles, walking frame, wheelchair, pressure relief cushions); glasses or contact lenses; other sensory and communications tools (screen reader, braille, hearing aids, sign language, communication boards); and others.

Healthcare expenditure at the household level was also measured as a secondary outcome. This was assessed by asking if anyone in the household had spent money on medicines (including anti-worming, cold tablets, vaccines, bandages, contraceptives, malarial medication, pain killers, and prescriptions), other medical/assistive devices (e.g., spectacles, contact lenses, hearing aid), doctor's consultation fees, hospital/clinic charges, traditional medicine, and other health expenditures in the last year. Unlike other health outcomes which were measured specifically for the index person with disabilities, health expenditure was measured at the household level for all members, as household finances are typically pooled in this context.

### Statistical analysis

Sample size was primarily based on the main trial's primary outcome of per-capita household expenditure as detailed in the published protocol.^32^ With 370 index persons in the intervention group and 321 in the control group at baseline, our study had 80% power to detect a standardized mean difference of 0.2 (indicating either a 0.2 increase or 0.2 decrease) for continuous outcomes and an odds ratio of 1.2 (indicating increased risk) or 0.83 (indicating decreased risk) for categorical outcomes, using a two-tailed test at 5% significance level. Power calculations were performed using the cps.binary function from the clusterPower package (version 0.7.0) in R, which employs Monte Carlo methods (simulations) to estimate power for cluster-randomized trials. Intra-class correlation coefficients (ICCs) were estimated from baseline data to account for potential clustering effects.

Analyses followed CONSORT guidelines using R version 4.0.1.

For both follow-up periods, we used linear mixed-effects regression with restricted maximum likelihood estimation for continuous outcomes, reporting minimally-adjusted mean differences (MAMDs), fully-adjusted mean differences (FAMDs), and standardized mean differences (using Hedges' method^37^) with 95% CIs. For binary outcomes, we used generalized estimating equations to estimate minimally-adjusted odds ratios (MAORs) and fully-adjusted odds ratios (FAORs) with 95% CIs, using Kauermann-Carroll bias-corrected standard errors to account for small cluster numbers.^38^

Minimally-adjusted models included treatment status as a fixed effect and random intercepts for cluster and branch. Fully-adjusted models additionally included variables showing imbalance (p < 0.10) at baseline or due to loss to follow-up. For binary outcomes, we used an exchangeable working correlation matrix to account for clustering.

To explore sex differences, we repeated analyses including an interaction term between treatment status and sex.

All analyses were prespecified and overseen by an independent Data Safety and Monitoring Board. The trial was registered with RIDIE (RIDIE-STUDY-ID-626008898983a) and ISRCTN (ISRCTN-78592382).

### Role of the funding source

The funder of the study had no role in study design, data collection, data analysis, data interpretation, or writing of the article.

## Results

Between January and March 2020, we screened 185 clusters across 164 villages, randomly allocating 96 clusters to intervention (DIG) and 89 to control (Fig. 1). At baseline, we identified 511 households with disabilities from intervention clusters and 434 from control clusters. Among these, 370 working-age person with disabilities (intervention) and 321 (control) were selected as index person and completed participant questionnaires. At first follow-up, 647 (93.6%) index person were successfully interviewed: 357 (96.5%) from intervention and 290 (90.3%) from control groups. At second follow-up, 643 (93.1%) index person were successfully interviewed: 354 (95.7%) from intervention and 289 (90.3%) from control groups.

Among index person interviewed at baseline, mean age was 35.71 years (SD 12.42) in intervention and 34.31 years (SD 11.97) in control groups. Female proportion was similar between groups (intervention: 53.5%, control: 53.6%). Baseline characteristics were largely balanced between groups (Table 1), though intervention group project participants were less likely to be married/cohabiting (53.5% vs 62.1%, p = 0.05) (Supplementary Table S1). Analysis of participants lost to first follow-up revealed that these households had significantly younger participants (p < 0.01) and index persons with lower reported anxiety (p = 0.09) (Supplementary Table S2). Participants lost to second follow-up were characterized by significantly younger age (p = 0.02), higher education levels (p = 0.10), and greater household income (p = 0.10) compared to those retained in the study (Supplementary Table S3).

**Table 1:** Baseline characteristics of index people with disabilities in the DIG intervention and control groups.

	DIG Intervention group (n = 370)	Control group (n = 321)	p value
**Individual-level factors for index person**			
**Age (years)**	35.7 (12.4)	34.3 (12.0)	0.14
**Sex (=female)**	198 (53.5%)	172 (53.6%)	1.00
**Level of education**			
No education	82 (22.2%)	77 (24.0%)	0.19
Primary education	226 (61.1%)	208 (64.8%)	
Secondary education	56 (15.1%)	31 (9.7%)	
Specialized training/bachelor or above	6 (1.6%)	5 (1.6%)	
**Marital status**			
Never married	123 (33.2%)	110 (34.3%)	0.13
Married/cohabiting	156 (42.2%)	152 (47.4%)	
Divorced/separated/widow	91 (24.6%)	59 (18.4%)	
**Is household head (=yes)**	173 (46.8%)	141 (43.9%)	0.50
**Is project participant (=yes)**	176 (47.6%)	153 (47.7%)	1.00
**Symptom of anxiety (=yes)**	85 (23.0%)	58 (18.1%)	0.14
**Symptom of depression (yes)**	63 (17.0%)	41 (12.8%)	0.15
**Having any illness or injury (=yes)**	193 (52.2%)	159 (49.5%)	0.54
**Having any unmet health need (=yes)**	45 (12.2%)	34 (10.6%)	0.60
**Health expenditure per capita (dollars) per month**	4.98 (6.40)	4.85 (6.03)	0.79
**Household-level factors**			
**Highest level of education**			
No education	3 (0.8%)	5 (1.6%)	0.64
Primary education	206 (55.7%)	188 (58.6%)	
Secondary education	140 (37.8%)	110 (34.3%)	
Specialized training/bachelor or above	21 (5.7%)	18 (5.6%)	
**Lives in poverty (=yes)**	370 (100.0%)	321 (100.0%)	–
**Household size**	5.8 (2.2)	5.8 (2.4)	0.93
Number of children in household	0.7 (0.9)	0.8 (1.0)	0.29
**Per capita income (dollars) per month**	69.3 (80.1)	66.6 (75.2)	0.64

Data was reported as the mean (standard deviation) or number (percentage). DIG, Disability-inclusive graduation programme.

At first follow-up (Table 2), the primary outcome of illness/injury was reported by 41.18% of intervention and 45.86% of control participants, showing no significant difference (MAOR 0.83, 95% CI 0.57–1.21, p = 0.34; FAOR 0.84, 95% CI 0.58–1.22, p = 0.36). No significant differences were found for most specific conditions: respiratory (FAOR 0.82, 95% CI 0.43–1.54, p = 0.53), gastrointestinal (FAOR 0.81, 95% CI 0.29–2.23, p = 0.68), or chronic conditions (FAOR 1.48, 95% CI 0.81–2.71, p = 0.20). Reported malaria showed a marginally lower trend in the intervention group (FAOR 0.65, 95% CI 0.40–1.06, p = 0.09). Mental well-being outcomes showed no significant differences in potential moderate depression (PHQ ≥10; FAOR 0.95, 95% CI 0.58–1.56, p = 0.84) or severe depression (PHQ ≥15; FAOR 2.23, 95% CI 0.78–6.40, p = 0.14). Additionally, the intervention group showed a marginally lower likelihood of reporting unmet health needs (FAOR 0.56, 95% CI 0.31–1.02, p = 0.06). Health expenditure per capita change from baseline showed no significant difference between groups (FAMD 0.69, 95% CI −0.30 to 1.68, p = 0.18; effect size = 0.13, 95% CI −0.06 to 0.33).

**Table 2:** Health effects of the Disability-inclusive graduation programme.

Outcomes	DIG intervention	Control group	Minimally adjusted analysis			Fully adjusted analysis		
			Odds ratio (95% CI)	Mean difference/Effect size (95% CI)	p value	Odds ratio (95% CI)	Mean difference/Effect size (95% CI)	p value
**First follow-up (0 month after the intervention)**								
**Having any illness or injury**	147 (41.18%)	133 (45.86%)	0.83 [0.57, 1.21]	–	0.34	0.84 [0.58, 1.22]	–	0.36
**Respiratory conditions**	22 (6.16%)	21 (7.24%)	0.84 [0.44, 1.59]	–	0.6	0.82 [0.43, 1.54]	–	0.53
**Gastrointestinal conditions**	6 (1.68%)	6 (2.07%)	0.78 [0.28, 2.23]	–	0.65	0.81 [0.29, 2.23]	–	0.68
**Malaria**	62 (17.37%)	70 (24.14%)	0.63 [0.39, 1.03]	–	0.06	0.65 [0.4, 1.06]	–	0.09
**Chronic conditions**	35 (9.8%)	22 (7.59%)	1.32 [0.74, 2.33]	–	0.35	1.48 [0.81, 2.71]	–	0.2
**Pain-related conditions**	53 (14.85%)	40 (13.79%)	1.17 [0.68, 2]	–	0.58	1.23 [0.72, 2.08]	–	0.45
**Injury**	13 (3.64%)	17 (5.86%)	0.58 [0.27, 1.25]	–	0.17	0.49 [0.22, 1.1]	–	0.08
**Mental well-being**								
Potential moderate depression (PHQ ≥10)	68 (19.05%)	53 (18.28%)	1.06 [0.65, 1.71]	–	0.82	0.95 [0.58, 1.56]	–	0.84
Potential severe depression (PHQ ≥15)	11 (3.08%)	6 (2.07%)	1.52 [0.54, 4.26]	–	0.43	2.23 [0.78, 6.4]	–	0.14
**Having any unmet health need**	22 (6.16%)	28 (9.66%)	0.61 [0.35, 1.07]	–	0.09	0.56 [0.31, 1.02]	–	0.06
**Health expenditure per capita, difference from baseline**	−0.61 (7.64)	−0.77 (6.88)	–	0.22 [−1.06, 1.51]/0.03 [−0.15, 0.21]	0.74	–	0.69 [−0.30, 1.68]/0.13 [−0.06, 0.33]	0.18
**Second follow-up (16 months after the intervention)**								
**Having any illness or injury**	197 (55.65%)	156 (53.98%)	1.08 [0.77, 1.52]	–	0.66	1.07 [0.74, 1.56]	–	0.71
**Respiratory conditions**	38 (10.73%)	35 (12.11%)	0.87 [0.49, 1.55]	–	0.64	0.79 [0.43, 1.45]	–	0.45
**Gastrointestinal conditions**	15 (4.24%)	8 (2.77%)	1.54 [0.62, 3.84]	–	0.35	1.67 [0.62, 4.47]	–	0.31
**Malaria**	90 (25.42%)	55 (19.03%)	1.46 [0.95, 2.24]	–	0.08	1.54 [0.97, 2.43]	–	0.07
**Chronic conditions**	30 (8.47%)	28 (9.69%)	0.86 [0.48, 1.55]	–	0.62	0.85 [0.47, 1.52]	–	0.58
**Pain-related conditions**	76 (21.47%)	62 (21.45%)	1.02 [0.65, 1.59]	–	0.95	1.05 [0.66, 1.68]	–	0.83
**Injury**	15 (4.24%)	9 (3.11%)	1.35 [0.57, 3.21]	–	0.5	1.36 [0.56, 3.33]	–	0.5
**Mental well-being**								
Potential moderate depression (PHQ ≥ 10)	91 (25.71%)	71 (24.57%)	1.07 [0.72, 1.61]	–	0.73	1.01 [0.65, 1.57]	–	0.97
Potential severe depression (PHQ ≥ 15)	26 (7.34%)	22 (7.61%)	0.94 [0.5, 1.78]	–	0.86	0.95 [0.49, 1.84]	–	0.88
**Having any unmet health need**	27 (7.63%)	43 (14.88%)	0.46 [0.26, 0.81]	–	**0.007**	0.4 [0.22, 0.71]	–	**0.002**
**Having any unmet assistive products or services**	143 (40.4%)	127 (43.94%)	0.91 [0.63, 1.31]	–	0.6	0.9 [0.62, 1.31]	–	0.59
Mobility Aids	42 (11.86%)	46 (15.92%)	0.71 [0.43, 1.15]	–	0.16	0.65 [0.4, 1.05]	–	0.08
Glasses or contact lenses	137 (38.7%)	131 (45.33%)	0.82 [0.55, 1.22]	–	0.33	0.84 [0.56, 1.27]	–	0.41
Other sensory and communications tools	5 (1.41%)	8 (2.77%)	0.53 [0.18, 1.57]	–	0.25	0.51 [0.18, 1.44]	–	0.2
Others	24 (6.78%)	18 (6.23%)	1.12 [0.58, 2.18]	–	0.73	1.11 [0.56, 2.23]	–	0.76
**Health expenditure per capita, difference from baseline**	−0.07 (7.72)	−0.78 (6.81)	–	0.69 [−0.72, 2.08]/0.09 [−0.1, 0.28]	0.33	–	0.93 [−0.22, 2.09]/0.18 [−0.04, 0.4]	0.12

Intervention effects were estimated using two statistical approaches: (1) generalized estimating equations reporting odds ratios with 95% confidence intervals (CIs) for binary outcomes, and (2) linear mixed-effects regression reporting mean differences and standardized effect sizes with 95% CIs for continuous outcomes. The minimally-adjusted model included treatment status (fixed effect) and cluster/branch (random intercepts). Fully adjusted models additionally controlled for imbalanced variables (p < 0.10): for first follow-up, including marital status and age of project participants, and symptom of anxiety of index person; for second follow-up including marital status and age of project participants, and household per capita income. PHQ, Patient Health Questionnaire.

At second follow-up (Table 2), the primary outcome of illness/injury was reported by 55.65% of intervention and 53.98% of control participants, showing no significant difference (MAOR 1.08, 95% CI 0.77–1.52, p = 0.657; FAOR 1.07, 95% CI 0.74–1.56, p = 0.71). No significant differences were found for most specific conditions: respiratory (FAOR 0.79, 95% CI 0.43–1.45, p = 0.45), gastrointestinal (FAOR 1.67, 95% CI 0.62–4.47, p = 0.31), or chronic conditions (FAOR 0.85, 95% CI 0.47–1.52, p = 0.58). Reported malaria was marginally higher in the intervention group (FAOR 1.54, 95% CI 0.97–2.43, p = 0.07). Mental well-being outcomes showed no significant differences in potential moderate depression (PHQ ≥10; FAOR 1.01, 95% CI 0.65–1.57, p = 0.97) or severe depression (PHQ ≥15; FAOR 0.95, 95% CI 0.49–1.84, p = 0.88). The intervention group had significantly lower likelihood of reporting unmet health needs (FAOR 0.4, 95% CI 0.22–0.71, p = 0.002). No significant differences were found for unmet assistive products/services overall (FAOR 0.9, 95% CI 0.62–1.31, p = 0.59), or for some specific categories such as glasses/contact lenses (FAOR 0.84, 95% CI 0.56–1.27, p = 0.41) or other sensory/communication tools (FAOR 0.51, 95% CI 0.18–1.44, p = 0.20). Unmet mobility aid needs showed marginal improvement (FAOR 0.65, 95% CI 0.4–1.05, p = 0.08).Health expenditure per capita change from baseline showed no significant difference between groups (FAMD 0.93, 95% CI −0.22 to 2.09, p = 0.12; effect size = 0.18, 95% CI −0.04 to 0.4).

Few sex-specific effects were observed (Table 3). In the second follow-up, intervention group females showed higher likelihood of pain-related conditions (FAOR for interaction 2.43, 95% CI 1.05–5.59, p = 0.04) but lower injury likelihood (FAOR for interaction 0.17, 95% CI 0.04–0.74, p = 0.02) compared to males. No significant sex differences were found for other outcomes including respiratory conditions, gastrointestinal conditions, chronic conditions, mental well-being measures, and unmet needs. The effects by sex and by follow-up were presented in Supplementary Table S4 and Supplementary Table S5.

**Table 3:** Sex difference on the health effects of the Disability-inclusive graduation programme, with male as the reference.

Outcomes	Male		Female		Minimally adjusted analysis			Fully adjusted analysis		
	DIG intervention	Control group	DIG intervention	Control group	Odds ratio (95% CI)	Mean difference/Effect size (95% CI)	p value	Odds ratio (95% CI)	Mean difference/Effect size (95% CI)	p value
**First follow-up (0 month after the intervention)**										
**Having any illness or injury**	61 (39.35%)	52 (41.27%)	86 (42.57%)	81 (49.39%)	0.83 [0.47, 1.45]	–	0.51	0.69 [0.38, 1.25]	–	0.22
**Respiratory conditions**	7 (4.52%)	10 (7.94%)	15 (7.43%)	11 (6.71%)	1.99 [0.52, 7.68]	–	0.32	1.78 [0.45, 7.02]	–	0.41
**Gastrointestinal conditions**	3 (1.94%)	1 (0.79%)	3 (1.49%)	5 (3.05%)	0.26 [0.03, 2.3]	–	0.22	0.26 [0.03, 2.05]	–	0.2
**Malaria**	23 (14.84%)	23 (18.25%)	39 (19.31%)	47 (28.66%)	0.72 [0.35, 1.51]	–	0.39	0.53 [0.24, 1.2]	–	0.13
**Chronic conditions**	18 (11.61%)	7 (5.56%)	17 (8.42%)	15 (9.15%)	0.43 [0.15, 1.17]	–	0.1	0.48 [0.16, 1.45]	–	0.19
**Pain-related conditions**	21 (13.55%)	18 (14.29%)	32 (15.84%)	22 (13.41%)	1.42 [0.58, 3.46]	–	0.44	1.42 [0.55, 3.71]	–	0.47
**Injury**	5 (3.23%)	5 (3.97%)	8 (3.96%)	12 (7.32%)	0.61 [0.18, 2.09]	–	0.44	0.49 [0.12, 2.02]	–	0.32
**Mental well-being**										
**Potential moderate depression (PHQ ≥10)**	28 (18.06%)	22 (17.46%)	40 (19.8%)	31 (18.9%)	1.09 [0.46, 2.58]	–	0.85	0.95 [0.38, 2.39]	–	0.91
**Potential severe depression (PHQ ≥15)**	5 (3.23%)	1 (0.79%)	6 (2.97%)	5 (3.05%)	0.32 [0.05, 1.87]	–	0.2	0.51 [0.07, 3.43]	–	0.49
**Having any unmet health need**	10 (6.45%)	10 (7.94%)	12 (5.94%)	18 (10.98%)	0.65 [0.2, 2.07]	–	0.46	0.5 [0.14, 1.8]	–	0.29
**Health expenditure per capita, difference from baseline**	−1.11 (7.99)	−0.95 (7.73)	−0.19 (7.35)	−0.63 (6.13)	–	0.68 [−1.73, 3.09]/0.09 [−0.24, 0.42]	0.58	–	1.14 [−0.54, 2.82]/0.22 [−0.11, 0.55]	0.18
**Second follow-up (16 months after the intervention)**										
**Having any illness or injury**	82 (52.23%)	72 (53.33%)	115 (58.38%)	84 (54.55%)	1.19 [0.65, 2.18]	–	0.57	1.15 [0.59, 2.25]	–	0.68
**Respiratory conditions**	16 (10.19%)	18 (13.33%)	22 (11.17%)	17 (11.04%)	1.3 [0.54, 3.09]	–	0.56	1.11 [0.4, 3.05]	–	0.84
**Gastrointestinal conditions**	6 (3.82%)	1 (0.74%)	9 (4.57%)	7 (4.55%)	0.26 [0.04, 1.82]	–	0.17	0.41 [0.05, 3.12]	–	0.39
**Malaria**	38 (24.2%)	23 (17.04%)	52 (26.4%)	32 (20.78%)	0.88 [0.42, 1.81]	–	0.72	0.93 [0.4, 2.13]	–	0.86
**Chronic conditions**	10 (6.37%)	10 (7.41%)	20 (10.15%)	18 (11.69%)	1.05 [0.35, 3.18]	–	0.93	1.02 [0.34, 3.11]	–	0.97
**Pain-related conditions**	25 (15.92%)	33 (24.44%)	51 (25.89%)	29 (18.83%)	2.43 [1.22, 4.84]	–	**0.01**	2.43 [1.05, 5.59]	–	**0.04**
**Injury**	7 (4.46%)	3 (2.22%)	8 (4.06%)	6 (3.9%)	0.53 [0.13, 2.12]	–	0.37	0.17 [0.04, 0.74]	–	**0.02**
**Mental well-being**										
Potential moderate depression (PHQ ≥10)	37 (23.57%)	29 (21.48%)	54 (27.41%)	42 (27.27%)	0.95 [0.46, 1.98]	–	0.89	1.15 [0.54, 2.42]	–	0.72
Potential severe depression (PHQ ≥15)	14 (8.92%)	8 (5.93%)	12 (6.09%)	14 (9.09%)	0.45 [0.15, 1.33]	–	0.15	0.56 [0.18, 1.73]	–	0.31
**Having any unmet health need**	11 (7.01%)	22 (16.3%)	16 (8.12%)	21 (13.64%)	1.38 [0.51, 3.74]	–	0.53	1.35 [0.46, 3.97]	–	0.59
**Having unmet assistive products or services**	55 (35.03%)	53 (39.26%)	88 (44.67%)	74 (48.05%)	1.09 [0.59, 1.99]	–	0.79	1.36 [0.68, 2.69]	–	0.38
Mobility aids	16 (10.19%)	19 (14.07%)	26 (13.2%)	27 (17.53%)	1.02 [0.45, 2.31]	–	0.97	1.22 [0.48, 3.12]	–	0.68
Glasses or contact lenses	51 (32.48%)	57 (42.22%)	86 (43.65%)	74 (48.05%)	1.37 [0.73, 2.58]	–	0.33	1.56 [0.78, 3.11]	–	0.21
Other sensory and communications tools	0 (0%)	4 (2.96%)	5 (2.54%)	4 (2.6%)	–		–	–		–
Others	14 (8.92%)	9 (6.67%)	10 (5.08%)	9 (5.84%)	0.65 [0.15, 2.73]	–	0.55	0.81 [0.2, 3.32]	–	0.77
**Health expenditure per capita, difference from baseline**	−0.28 (7.41)	−1.51 (7.98)	0.1 (7.98)	−0.14 (5.53)	–	−1.03 [−3.48, 1.43]/−0.14 [−0.48, 0.2]	0.41	–	−0.18 [−1.91, 1.55]/−0.03 [−0.36, 0.29]	0.84

Intervention effects were estimated using two statistical approaches: (1) generalized estimating equations reporting odds ratios with 95% confidence intervals (CIs) for binary outcomes, and (2) linear mixed-effects regression reporting mean differences and standardized effect sizes with 95% CIs for continuous outcomes. The minimally-adjusted model included treatment status (fixed effect) and cluster/branch (random intercepts). Fully adjusted models additionally controlled for imbalanced variables (p < 0.10): marital status and age of project participants, and household per capita income.

## Discussion

To our knowledge, this study is the first cluster randomized trial to evaluate the effectiveness of a disability-inclusive graduation (DIG) programme on health outcomes among ultra-poor households with disabilities in a low-income setting. We found no significant impact of the intervention on the primary outcome—illness or injury prevalence—at either first or second follow-up, though the overall prevalence increased in both groups over time. However, the intervention showed notable effects on some secondary outcomes. The impact on unmet health needs strengthened over time, with marginally lower likelihood at first follow-up becoming significantly lower by second follow-up, indicating progressive improvement in healthcare access. Additionally, while there was no significant impact on unmet assistive products or services overall, the likelihood of having unmet needs for mobility aids was marginally lower in the intervention group, suggesting a potential trend towards improved access in this area. The intervention's effect on malaria showed an interesting pattern, shifting from marginally lower likelihood at first follow-up to marginally higher at second follow-up, suggesting potential changes in risk exposure over time. No significant differences were observed for mental health outcomes or health expenditure per capita at either time point. We also found that sex modified the intervention's effects. Females in the intervention group were more likely to experience pain-related conditions but less likely to report injuries compared to males by second follow-up.

The successful implementation of this complex intervention in Northern Uganda demonstrates that disability-inclusive graduation programs are feasible in low-income settings when delivered with appropriate infrastructure and partnerships. By leveraging existing community structures and collaborating with established organizations (BRAC, HI, and NUWODU), the intervention achieved high fidelity and retention rates despite resource constraints. While such comprehensive programs require significant investment, the model's integrated approach potentially offers greater efficiency than fragmented service delivery. The progressive strengthening of effects on healthcare access provides encouraging evidence for the value of such investments, though the need for enhanced health-specific components remains evident. Future implementations should consider cost-efficiency analyses to optimize resource allocation while maintaining effectiveness.

The lack of significant impact on illness/injury prevalence at both follow-up points, with prevalence actually increasing over time in both groups, aligns with mixed evidence from poverty alleviation programs. As this constituted our primary outcome and demonstrated no significant intervention effect, we interpret findings from secondary outcomes and subgroup analyses with appropriate circumspection. While some studies show positive health impacts for specific conditions,^16^^,^^21^ others report limited effects on general health status.^18^ Our findings suggest that the relationship between economic empowerment and health outcomes may be more complex for people with disabilities, potentially requiring longer intervention periods or more targeted health components to achieve measurable improvements in overall health status. Nevertheless, the observed increase in illness/injury prevalence across both groups warrants contextual interpretation, as it coincided with the COVID-19 pandemic. Direct infections, disrupted healthcare services, delayed care for chronic non-communicable diseases (NCDs), and pandemic-related mental health impacts likely contributed to these patterns. These extraordinary circumstances may have attenuated the intervention's potential health impacts while highlighting the importance of the improved healthcare access achieved through the program.

The evolution of reduced unmet health needs—from marginally significant at first follow-up to strongly significant at second follow-up—provides compelling evidence of the DIG program's progressive impact on healthcare access. This strengthening effect over time aligns with evidence from disability-targeted interventions in China and Kenya, where improved healthcare utilization was observed among disability allowance recipients.^26^^,^^28^ The intensifying impact suggests that the DIG program's comprehensive approach—combining cash transfers with emergency health funds, health education, and Community Health Promoter linkages—may require time to fully address multiple healthcare access barriers beyond financial constraints alone.

This finding reveals complex links between economic empowerment and health risks, indicating that poverty reduction programs should explicitly incorporate disease prevention strategies, especially for conditions affected by changing participants' circumstances activity patterns.

The contrasting temporal patterns in malaria likelihood—from marginally lower in the intervention group at first follow-up to marginally higher at second follow-up— suggest potential variation in the program's longer-term effects that warrants further investigation. Notingly, these finding represent exploratory ones that warrant cautious interpretation given the multiplicity of statistical comparisons. This shift could reflect the gradual impact of changing economic activities and daily routines stemming from the program's livelihood components. The temporal shift in malaria prevalence patterns between follow-up periods suggests complex interactions between the intervention and malaria risk factors that warrant further investigation. While our data cannot establish causal mechanisms for these observations, they highlight the importance of monitoring potential unintended consequences of economic empowerment interventions on disease exposure patterns. This finding highlights the need for ongoing health risk assessment and mitigation strategies if poverty reduction programs change participants' activity patterns.

The consistently negligible impact on mental health outcomes and health expenditure across both follow-up periods contrasts with previous literature. While earlier graduation programs showed improved psychological well-being, particularly in Bangladesh,^31^ our different findings may reflect that mental health challenges among people with disabilities stem from complex interactions of impairment-related factors, societal barriers, and discrimination,^1^ may require targeted interventions beyond economic empowerment. The persistent lack of impact on health expenditure, contrary to Chinese studies showing increased healthcare spending from disability-targeted cash transfers,^26^^,^^27^ likely reflects the DIG program's sustained effectiveness in providing direct health supports that reduce out-of-pocket expenditure needs.

Despite assistive technology provision being a key component of the DIG programme, the differential impact on assistive technology needs–marginal improvements in mobility aid access but no overall change in assistive product needs–illuminates the complexities of addressing assistive technology access through poverty reduction programs. The limited success of this programme component, particularly for comprehensive assistive technology provision, partially aligns with evidence that financial barriers are just one of multiple obstacles.^1^ The marginally positive effect on mobility aids likely reflects their greater local market availability compared to products like hearing aids or communication devices, making them more responsive to increased financial resources. However, the lack of impact on other assistive products suggests persistent barriers beyond financial constraints, including limited availability, complex procurement processes, and insufficient technical expertise for fitting and maintenance. This apparent failure of the DIG programme to fully deliver on its assistive technology objectives demonstrates that embedding assistive technology provision within poverty reduction programmes may be insufficient. While comprehensive poverty reduction programs may improve access to readily available devices like mobility aids, more specialized and targeted interventions, with dedicated technical expertise and procurement systems, are needed to effectively deliver the full spectrum of assistive products. These findings suggest a critical need to reassess and strengthen how assistive technology provision is integrated into graduation programmes.

The pattern of progressively improving healthcare access without corresponding health status improvements provides important insights about intervention timeframes. The strengthening effect on unmet health needs suggests that the program successfully addresses immediate healthcare access barriers, but the lag in measurable health benefits may indicate that longer periods are needed to translate improved access into better health outcomes. This aligns with previous studies showing that health benefits of poverty reduction programs often manifest over extended timeframes.^29^

The observed sex differences in health outcomes by second follow-up—with higher prevalence of pain-related conditions but lower injury rates among females— suggest possible temporal aspects of gender dynamics in poverty reduction programs that merit additional study. These findings should also be considered hypothesis-generating ones rather than definitive effects, particularly in the context of multiple comparisons and the absence of effect on the primary outcome. The higher prevalence of pain-related conditions among females might reflect differing physical demands of DIG program livelihood activities, potentially increasing musculoskeletal strain, while lower female injury rates likely reflect gender differences in risk exposure, with men potentially engaging in more hazardous work. These patterns align with literature showing women with disabilities face double discrimination and may be channelled into specific economic activities that exacerbate certain health conditions.^39^^,^^40^ The differential impacts underscore the importance of long-term monitoring and gender-sensitive program adjustments to address emerging health risks while maintaining beneficial protective effects, particularly as participants engage in new economic activities over time. These sex-differentiated effects emphasize the need to consider gender when designing and evaluating disability-inclusive poverty reduction programs to address distinct health risks and needs of male and female participants.

Our findings align with the social model of disability, where disability arises from the interaction between individuals with impairments and environmental barriers.^41^ While the intervention addressed individual-level factors through rehabilitation and assistive devices, the lack of improvement in overall health outcomes suggests that broader environmental barriers in Northern Uganda—limited healthcare infrastructure, geographical inaccessibility, and social stigma—may have constrained the program's health impact. Future disability-inclusive interventions should consider more explicitly targeting these environmental barriers to create enabling conditions that support better health outcomes for people with disabilities.

Our findings have important implications for policy, practice, and future research. While acknowledging the exploratory nature of many of our findings in the context of multiple statistical comparisons, the progressively strengthening effect on reducing unmet health needs—from marginal to significant improvement—suggests that comprehensive poverty reduction programmes can improve healthcare access for people with disabilities over time, supporting the integration of disability-specific health components within broader poverty reduction initiatives. The contrasting temporal patterns in malaria risk highlight the need for ongoing health risk assessment as economic circumstances change. Similarly, while the marginal reduction in unmet needs for mobility aids by second follow-up suggests that graduation programmes may gradually help overcome financial barriers for some assistive products, the lack of improvement in other assistive technology needs indicates that additional policy measures are needed to address persistent access barriers. Moreover, the AT component of the DIG programme clearly needs to be strengthened. The emergence of sex-differentiated effects by second follow-up underscores the importance of sustained monitoring and gender-sensitive programme design, while the consistently negligible impact on mental health outcomes and most physical health conditions across both follow-up periods suggests that longer-term support or more intensive health-specific interventions may be necessary. These findings collectively suggest that while comprehensive poverty reduction programmes can improve some aspects of healthcare access for people with disabilities, achieving broader health improvements requires additional actions to overcome barriers to healthcare seeking (e.g., health promotion, gender considerations), alongside careful attention to evolving health risks and longer intervention periods, while maintaining the successful elements of the disability-inclusive graduation approach.^42^

This study has several key strengths. It represents one of the first cluster randomized controlled **trial**s examining health outcomes of a graduation programme for people with disabilities in a low-income setting, with cluster randomization minimizing contamination between groups while accounting for village-level intervention components. The inclusion of both first and second follow-up measurements allows examination of temporal patterns in intervention effects, revealing how impacts evolve over time. The comprehensive measurement of health outcomes–including physical illness/injury, mental health, healthcare access, and expenditure–provides a holistic impact assessment. Sex-stratified analyses revealed important differential effects between males and females, while high follow-up rates (∼95% intervention, ∼90% control) strengthen findings' validity.

However, several limitations should be considered when interpreting the results. First, a significant contextual limitation is that the study was conducted during the COVID-19 pandemic, which may have substantially impacted our results and potentially limits their generalizability. Lockdown measures, healthcare resource diversion, and economic disruptions in Uganda may have influenced both intervention delivery and outcome patterns. The increased illness/injury prevalence across both groups could partially reflect pandemic-related health impacts, while healthcare seeking behaviors were likely affected by COVID-19 restrictions and concerns. Second, the differential loss to follow-up between groups, with participants lost being younger, more educated, and having higher per capita income, may have introduced selection bias. Although our fully adjusted analyses attempted to account for these differences, some residual confounding may remain. Third, due to data collection equipment failure at first follow-up, we were unable to assess the intervention's initial impact on unmet assistive products or services needs, limiting our understanding of how these outcomes evolved over the full study period. Fourth, while the inclusion of two follow-up periods provided valuable temporal insights, these timeframes may still be insufficient to capture longer-term health effects of the intervention, particularly for chronic conditions or sustained behavioral changes in healthcare seeking. Fifth, some of our health outcome measures relied on self-reported data, which could be subject to recall bias or social desirability bias, especially given the inability to mask participants to their intervention status, which could have introduced performance bias.^43^ Sixth, the measurement of mental health status changed between baseline (Washington Group instrument) and follow-up (PHQ-9), limiting our ability to assess changes in mental health over time. Seventh, while our study included multiple health outcomes, some important aspects of health such as preventive care utilization or quality of care received were not captured. Moreover, health expenditure was measured at the household level rather than for the individual with disabilities. Eighth, another limitation is that our sample size was primarily determined based on the trial's economic outcomes. With 370 intervention and 321 control participants at baseline, our study had 80% power to detect standardized mean differences of approximately 0.2 (either positive or negative) for continuous outcomes and odds ratios of 1.2 (indicating increased risk) or 0.83 (indicating decreased risk) for categorical outcomes. Consequently, our analysis may have been underpowered to detect smaller but potentially meaningful health effects, particularly for marginally significant outcomes such as unmet health needs at first follow-up and mobility aid needs at second follow-up. Nineth, the study's focus on Northern Uganda, while important for understanding impacts in a post-conflict, resource-limited setting, may limit generalizability to other contexts with different healthcare systems or disability support services. Finally, our reliance on self-reported illness/injury and unmet health needs presents additional limitations. Respondents self-identified their conditions without clinical verification, potentially leading to misclassification of illnesses like malaria or typhoid that require laboratory confirmation for accurate diagnosis. These limitations point to several key directions for future research. Studies should evaluate similar programs in post-pandemic contexts to determine whether observed patterns persist under more typical conditions. Longer follow-up periods (3–5 years) would help determine if improvements in healthcare access eventually translate into better health outcomes. Incorporating objective health measures alongside self-reported data would address potential reporting biases. Future research should also explore how enhancing the DIG model with health-specific components might improve outcomes beyond healthcare access. Given the observed sex-differentiated effects, studies specifically examining gender-specific impacts would be valuable. Finally, comparative research across diverse settings would help determine the generalizability of our findings.

In summary, this cluster-randomized trial provides the first evidence on health impacts of a disability-inclusive graduation programme among ultra-poor people with disabilities, with findings from two follow-up periods revealing how effects evolved over time. While the intervention did not significantly affect overall illness/injury prevalence at either follow-up point, it demonstrated progressively improving healthcare access, with marginally lower unmet health needs at first follow-up strengthening to significant improvement by second follow-up. The temporal patterns in outcomes revealed complex relationships between economic empowerment and health, with some effects strengthening over time (healthcare access), others showing mixed patterns (contrasting malaria risk), and some remaining consistently unchanged (mental health outcomes). The findings also captured emerging gender differences in health impacts by second follow-up, highlighting the importance of sustained monitoring of sex-specific effects. These results suggest that while comprehensive poverty reduction programmes can improve healthcare access for people with disabilities, the pathway to broader health improvements may require longer intervention periods and careful attention to evolving health risks. Future disability-inclusive graduation programmes should consider incorporating enhanced disease prevention strategies, gender-sensitive design elements, and sustained health support while maintaining successful aspects of integrated poverty reduction approaches. Additionally, programs should plan for longer-term monitoring to fully capture health impacts that may take time to manifest, while maintaining flexibility to address emerging health risks as participants engage in new economic activities.

## Contributors

SC conducted the statistical analysis, interpreted the results, and wrote the first draft of the manuscript. LMB, EK, CD, and MS provided critical intellectual input, reviewed the analysis, and made substantial revisions to the manuscript. HK conceptualized the study, secured funding, and served as the principal investigator, providing overall leadership for the trial in collaboration with MS. LMB, EK, and CD contributed to the study design, development of the trial protocol, and methodology. Both SC and EK have full access to the data and have verified the underlying data. All authors reviewed and approved the final version of the manuscript and agree to be accountable for all aspects of the work.

## Data sharing statement

De-identified participant data will be made available upon reasonable request after the end of the project (anticipated May 2025). Interested researchers should contact the corresponding author to request access. Data will be shared in accordance with ethical approvals and institutional data sharing policies, and requests will be reviewed by the study team to ensure appropriate use.

## Declaration of interests

We declare no competing interests.

## Appendix A Supplementary data

Supplementary Tables and Figures

Protocol

## References

[1] WHO (2022).

[2] Smythe T., Kuper H. (2024). The association between disability and all-cause mortality in low-income and middle-income countries: a systematic review and meta-analysis. Lancet Glob Health.

[3] Kuper H., Rotenberg S., Azizatunnisa L., Banks L.M., Smythe T. (2024). The association between disability and mortality: a mixed-methods study. Lancet Public Health.

[4] Kuper H., Heydt P. (2019).

[5] Inititiative MBIaCHA (2022).

[6] Bright T., Kuper H. (2018). A systematic review of access to general healthcare services for people with disabilities in low and middle income countries. Int J Environ Res Public Health.

[7] Hashemi G., Wickenden M., Bright T., Kuper H. (2022). Barriers to accessing primary healthcare services for people with disabilities in low and middle-income countries, a Meta-synthesis of qualitative studies. Disabil Rehabil.

[8] Banks L.M., Mearkle R., Mactaggart I., Walsham M., Kuper H., Blanchet K. (2017). Disability and social protection programmes in low- and middle-income countries: a systematic review. Oxf Dev Stud.

[9] Dalal J., Mitra S., James A., Rivas Velarde M. (2024). Links across disabilities: unveiling associations between functional domains. BMC Public Health.

[10] Banks L.M., Kuper H., Polack S. (2017). Poverty and disability in low- and middle-income countries: a systematic review. PLoS One.

[11] Hanass-Hancock J., Nene S., Deghaye N., Pillay S. (2017). ‘These are not luxuries, it is essential for access to life': disability related out-of-pocket costs as a driver of economic vulnerability in South Africa. Afr J Disabil.

[12] Walsham M., Kuper H., Banks L.M., Blanchet K. (2018). Social protection for people with disabilities in Africa and Asia: a review of programmes for low- and middle-income countries. Oxf Dev Stud.

[13] O'Donnell O. (2024). Health and health system effects on poverty: a narrative review of global evidence. Health Policy.

[14] Banks L.M., Walsham M., Minh H.V. (2019). Access to social protection among people with disabilities: evidence from Viet Nam. Int Soc Secur Rev.

[15] Banks L.M., Walsham M., Neupane S. (2019). Access to social protection among people with disabilities: mixed methods research from Tanahun, Nepal. Eur J Dev Res.

[16] Guimarães N.S., Magno L., de Paula A.A. (2023). The effects of cash transfer programmes on HIV/AIDS prevention and care outcomes: a systematic review and meta-analysis of intervention studies. Lancet HIV.

[17] Shahidi F.V., Ramraj C., Sod-Erdene O., Hildebrand V., Siddiqi A. (2019). The impact of social assistance programs on population health: a systematic review of research in high-income countries. BMC Public Health.

[18] Pega F., Pabayo R., Benny C., Lee E.Y., Lhachimi S.K., Liu S.Y. (2022). Unconditional cash transfers for reducing poverty and vulnerabilities: effect on use of health services and health outcomes in low- and middle-income countries. Cochrane Database Syst Rev.

[19] Richterman A., Millien C., Bair E.F. (2023). The effects of cash transfers on adult and child mortality in low- and middle-income countries. Nature.

[20] Jacobs W., Downey L.E. (2022). Impact of conditional cash transfer programmes on antenatal care service uptake in low and middle-income countries: a systematic review. BMJ Open.

[21] Ranganathan M., Lagarde M. (2012). Promoting healthy behaviours and improving health outcomes in low and middle income countries: a review of the impact of conditional cash transfer programmes. Prev Med.

[22] Lagarde M., Haines A., Palmer N. (2009). The impact of conditional cash transfers on health outcomes and use of health services in low and middle income countries. Cochrane Database Syst Rev.

[23] Rogers K., Le Kirkegaard R., Wamoyi J., Grooms K., Essajee S., Palermo T. (2024). Systematic review of cash plus or bundled interventions targeting adolescents in Africa to reduce HIV risk. BMC Public Health.

[24] Yoshino C.A., Sidney-Annerstedt K., Wingfield T. (2023). Experiences of conditional and unconditional cash transfers intended for improving health outcomes and health service use: a qualitative evidence synthesis. Cochrane Database Syst Rev.

[25] Pullar J., Allen L., Townsend N. (2018). The impact of poverty reduction and development interventions on non-communicable diseases and their behavioural risk factors in low and lower-middle income countries: a systematic review. PLoS One.

[26] Wang H., Li Z., Chen S. (2023). The effect of a disability-targeted cash transfer program on universal health coverage and universal access to education: a nationwide cohort study of Chinese children and adolescents with disabilities. Lancet Reg Health West Pac.

[27] Li Z., Wang H., Chen S. (2023). The association of a disability-targeted cash transfer programme with disability status and health-care access: a quasi-experimental study using a nationwide cohort of 4·3 million Chinese adults living with severe disabilities. Lancet Public Health.

[28] Abdille I.K., Mbataru P. (2019). Cash transfer and the economic well-being of persons with severe disability in Wajir County, Kenya. Int J Curr Asp.

[29] Banerjee A., Duflo E., Goldberg N. (2015). Development economics. A multifaceted program causes lasting progress for the very poor: evidence from six countries. Science.

[30] Raza W.A., Van de Poel E., Van Ourti T. (2018). Impact and spill-over effects of an asset transfer program on child undernutrition: evidence from a randomized control trial in Bangladesh. J Health Econ.

[31] Bandiera O., Burgess R., Das N., Gulesci S., Rasul I., Sulaiman M. (2017). Labor markets and poverty in village economies. Q J Econ.

[32] Kipchumba E., Davey C., Marks S. (2024). Evaluation of a disability-inclusive ultra-poor graduation programme in Uganda: study protocol for a cluster-randomised controlled trial with process evaluation. Trials.

[33] de Castro F., Cappa C., Madans J. (2023). Anxiety and depression signs among adolescents in 26 low- and middle-income countries: prevalence and association with functional difficulties. J Adolesc Health.

[34] Loeb M., Mont D., Cappa C., De Palma E., Madans J., Crialesi R. (2018). The development and testing of a module on child functioning for identifying children with disabilities on surveys. I: background. Disabil Health J.

[35] Arnold B.F., Rerolle F., Tedijanto C. (2024). Geographic pair matching in large-scale cluster randomized trials. Nat Commun.

[36] Rahman Ankhi M., Shammah A., Macharia A., Scovia M. (2023).

[37] Hedges L.V. (2007). Effect sizes in cluster-randomized designs. J Educ Behav Stat.

[38] Kauermann G., Carroll R.J. (2001). A note on the efficiency of sandwich covariance matrix estimation. J Am Stat Assoc.

[39] Matin B.K., Williamson H.J., Karyani A.K., Rezaei S., Soofi M., Soltani S. (2021). Barriers in access to healthcare for women with disabilities: a systematic review in qualitative studies. BMC Womens Health.

[40] Women U. (2018).

[41] World Health Organization (2022).

[42] Chipanta D., Estill J., Stöckl H. (2024). Differences in condom access and use and associated factors between persons with and without disabilities receiving social cash transfers in Luapula province, Zambia-A cross-sectional study. PLoS One.

[43] Forbes D. (2013). Blinding: an essential component in decreasing risk of bias in experimental designs. Evid Based Nurs.

