# Supplementary material for: Evaluation of health impacts of a disability-inclusive graduation programme among ultra-poor people with disabilities in Uganda: secondary analysis of a cluster randomized trial
Source: eClinicalMedicine. 2025 Jul 3;85:103318. doi: 10.1016/j.eclinm.2025.103318 (PMC12270699; doi:10.1016/j.eclinm.2025.103318)
Supplement: Supplementary Tables and Figures [file mmc1.docx]

**Sup Table 1.** **Basic description of project participants.** Data was reported as the mean (standard deviation) or number (percentage). P values were extracted from two-tailed test for continuous variables and two-tailed chi-square test for categorical variables.

|  | **DIG Intervention group**  **(n = 370)** | **Control group**  **(n = 321)** | **p value** |
| --- | --- | --- | --- |
| **Age (years)** | 42.3 (14.3) | 41.6 (14.0) | 0.54 |
| **Sex (=female)** | 270 (73.0%) | 224 (69.8%) | 0.40 |
| **Level of education** |  |  |  |
| No education | 70 (18.9%) | 68 (21.2%) | 0.84 |
| Primary education | 250 (67.6%) | 210 (65.4%) |  |
| Secondary education | 45 (12.2%) | 40 (12.5%) |  |
| Specialized training/bachelor or above | 5 (1.4%) | 3 (0.9%) |  |
| **Marital status** |  |  |  |
| Never married | 35 (9.5%) | 31 (9.7%) | **0.05** |
| Married/cohabiting | 197 (53.5%) | 198 (62.1%) |  |
| Divorced/separated/widow | 136 (37.0%) | 90 (28.2%) |  |
| **Is household head (=yes)** | 231 (62.4%) | 188 (58.6%) | 0.34 |

**Sup Table 2.** **Basic description by loss to first follow-up.** Data was reported as the mean (standard deviation) or number (percentage). P values were extracted from two-tailed test for continuous variables and two-tailed chi-square test for categorical variables.

|  | **Loss to first follow-up** | | **p value** |
| --- | --- | --- | --- |
|  | Yes (n = 42) | No (n = 649) |  |
| **Individual-level factors for index person** |  |  |  |
| **Age (years)** | 35.2 (12.3) | 35.1 (12.2) | 0.95 |
| **Sex (=female)** | 23 (54.8%) | 347 (53.5%) | 1.00 |
| **Level of education** |  |  |  |
| No education | 8 (19.0%) | 151 (23.3%) | 0.90 |
| Primary education | 28 (66.7%) | 406 (62.6%) |  |
| Secondary education | 5 (11.9%) | 82 (12.6%) |  |
| Specialized training/bachelor or above | 1 (2.4%) | 10 (1.5%) |  |
| **Marital status** |  |  |  |
| Never married | 9 (21.4%) | 224 (34.5%) | 0.11 |
| Married/cohabiting | 25 (59.5%) | 283 (43.6%) |  |
| Divorced/separated/widow | 8 (19.0%) | 142 (21.9%) |  |
| **Is household head (=yes)** | 24 (57.1%) | 290 (44.7%) | 0.16 |
| **Is project participant (=yes)** | 21 (50.0%) | 308 (47.5%) | 0.87 |
| **Symptom of anxiety (=yes)** | 4 (9.5%) | 139 (21.4%) | 0.09 |
| **Symptom of depression (=yes)** | 7 (16.7%) | 97 (14.9%) | 0.94 |
| **Having any illness or injury (=yes)** | 19 (45.2%) | 333 (51.3%) | 0.55 |
| **Having any unmet health need (=yes)** | 6 (14.3%) | 136 (21.0%) | 0.40 |
| **Health expenditure per capita (dollars)** | 6.2 (6.8) | 4.8 (6.2) | 0.23 |
| **Individual-level factors for project Participants** |  |  |  |
| **Age (years)** | 35.1 (11.5) | 42.4 (14.2) | <0.01 |
| **Sex (=female)** | 29 (69.0%) | 465 (71.6%) | 0.85 |
| **Level of education** |  |  |  |
| No education | 5 (11.9%) | 133 (20.5%) | 0.46 |
| Primary education | 32 (76.2%) | 428 (65.9%) |  |
| Secondary education | 5 (11.9%) | 80 (12.3%) |  |
| Specialized training/bachelor or above | 0 (0%) | 8 (1.2%) |  |
| **Marital status** |  |  |  |
| Never married | 5 (11.9%) | 61 (9.5%) | 0.42 |
| Married/cohabiting | 27 (64.3%) | 368 (57.1%) |  |
| Divorced/separated/widow | 10 (23.8%) | 216 (33.5%) |  |
| **Is household head (=yes)** | 24 (57.1%) | 395 (60.9%) | 0.75 |
| **Household-level factors** |  |  |  |
| **Highest level of education** |  |  |  |
| No education | 0 (0%) | 8 (1.2%) | 0.84 |
| Primary education | 25 (59.5%) | 369 (56.9%) |  |
| Secondary education | 14 (33.3%) | 236 (36.4%) |  |
| Specialized training/bachelor or above | 3 (7.1%) | 36 (5.5%) |  |
| **Lives in poverty (=yes)** | 39 (92.9%) | 616 (94.9%) | 0.82 |
| **Household size** | 5.7 (1.9) | 5.8 (2.3) | 0.77 |
| **Per capita income (dollars) per month** | 73.8 (78.1) | 67.7 (77.8) | 0.62 |

**Sup Table 3.** **Basic description by loss to second follow-up.** Data was reported as the mean (standard deviation) or number (percentage). P values were extracted from two-tailed test for continuous variables and two-tailed chi-square test for categorical variables.

|  | **Loss to second follow-up** | | **p value** |
| --- | --- | --- | --- |
|  | Yes (44) | No (647) |  |
| **Individual-level factors for index person** |  |  |  |
| **Age (years)** | 33.1 (10.6) | 35.2 (12.3) | 0.20 |
| **Sex (=female)** | 24 (54.5%) | 346 (53.5%) | 1.00 |
| **Level of education** |  |  |  |
| No education | 10 (22.7%) | 149 (23.0%) | 0.83 |
| Primary education | 29 (65.9%) | 405 (62.6%) |  |
| Secondary education | 5 (11.4%) | 82 (12.7%) |  |
| Specialized training/bachelor or above | 0 (0%) | 11 (1.7%) |  |
| **Marital status** |  |  |  |
| Never married | 12 (27.3%) | 221 (34.2%) | 0.54 |
| Married/cohabiting | 23 (52.3%) | 285 (44.0%) |  |
| Divorced/separated/widow | 9 (20.5%) | 141 (21.8%) |  |
| **Is household head (=yes)** | 24 (54.5%) | 290 (44.8%) | 0.27 |
| **Is project participant (=yes)** | 20 (45.5%) | 309 (47.8%) | 0.89 |
| **Symptom of anxiety (=yes)** | 11 (25.0%) | 132 (20.4%) | 0.59 |
| **Symptom of depression (=yes)** | 5 (11.4%) | 99 (15.3%) | 0.62 |
| **Having any illness or injury (=yes)** | 21 (47.7%) | 331 (51.2%) | 0.78 |
| **Having any unmet health need (=yes)** | 9 (20.5%) | 133 (20.6%) | 1.00 |
| **Health expenditure per capita (dollars)** | 5.6 (6.3) | 4.9 (6.2) | 0.49 |
| **Individual-level factors for project Participants** |  |  |  |
| **Age (years)** | 37.5 (12.1) | 42.3 (14.3) | 0.02 |
| **Sex (=female)** | 31 (70.5%) | 463 (71.6%) | 1.00 |
| **Level of education** |  |  |  |
| No education | 6 (13.6%) | 132 (20.4%) | 0.10 |
| Primary education | 29 (65.9%) | 431 (66.6%) |  |
| Secondary education | 7 (15.9%) | 78 (12.1%) |  |
| Specialized training/bachelor or above | 2 (4.5%) | 6 (0.9%) |  |
| **Marital status** |  |  |  |
| Never married | 5 (11.4%) | 61 (9.5%) | 0.51 |
| Married/cohabiting | 28 (63.6%) | 367 (57.1%) |  |
| Divorced/separated/widow | 11 (25.0%) | 215 (33.4%) |  |
| **Is household head (=yes)** | 25 (56.8%) | 394 (60.9%) | 0.71 |
| **Household-level factors** |  |  |  |
| **Highest level of education** |  |  |  |
| No education | 1 (2.3%) | 7 (1.1%) | 0.44 |
| Primary education | 29 (65.9%) | 365 (56.4%) |  |
| Secondary education | 13 (29.5%) | 237 (36.6%) |  |
| Specialized training/bachelor or above | 1 (2.3%) | 38 (5.9%) |  |
| **Lives in poverty (=yes)** | 40 (90.9%) | 615 (95.1%) | 0.40 |
| **Household size** | 5.8 (2.1) | 5.8 (2.3) | 0.95 |
| **Per capita income (dollars) per month** | 85.2 (72.6) | 66.2 (77.5) | 0.10 |

**Sup Table 4. Health effects of the Disability-inclusive graduation programme on females with disabilities.** Intervention effects were estimated using two statistical approaches: (1) generalized estimating equations reporting odds ratios with 95% confidence intervals (CIs) for binary outcomes, and (2) linear mixed-effects regression reporting mean differences and standardized effect sizes with 95% CIs for continuous outcomes. The minimally-adjusted model included treatment status (fixed effect) and cluster/branch (random intercepts). Fully adjusted models additionally controlled for imbalanced variables (p<0.10): for first follow-up, including marital status and age of project participants, and symptom of anxiety of index person; for second follow-up including marital status and age of project participants, and household per capita income. †, result was reported as mean difference and its 95% CI.

| **Outcomes** | **Minimally adjusted analysis** | | **Effect size (95% CI)** | **Fully adjusted analysis** | | **Effect size (95% CI)** |
| --- | --- | --- | --- | --- | --- | --- |
|  | **Mean difference or odds ratio (95% CI)** | **p value** |  | **Mean difference or odds ratio (95% CI)** | **p value** |  |
| **First follow-up (0 month after the intervention)** | | | | | | |
| **Having any illness or injury** | 0.76 [0.48, 1.2] | 0.24 | ·· | 0.84 [0.58, 1.22] | 0.36 | ·· |
| **Respiratory conditions** | 1.19 [0.52, 2.72] | 0.67 | ·· | 0.82 [0.43, 1.54] | 0.53 | ·· |
| **Gastrointestinal conditions** | 0.49 [0.14, 1.8] | 0.28 | ·· | 0.81 [0.29, 2.23] | 0.68 | ·· |
| **Malaria** | 0.57 [0.33, 0.97] | 0.04 | ·· | 0.65 [0.4, 1.06] | 0.09 | ·· |
| **Chronic conditions** | 0.92 [0.45, 1.92] | 0.83 | ·· | 1.48 [0.81, 2.71] | 0.20 | ·· |
| **Pain-related conditions** | 1.28 [0.67, 2.45] | 0.45 | ·· | 1.23 [0.72, 2.08] | 0.45 | ·· |
| **Injury** | 0.52 [0.21, 1.28] | 0.15 | ·· | 0.49 [0.22, 1.1] | 0.08 | ·· |
| **Mental well-being** |  |  | ·· |  |  | ·· |
| Potential moderate depression (PHQ>=10) | 1.1 [0.58, 2.12] | 0.76 | ·· | 0.95 [0.58, 1.56] | 0.84 | ·· |
| Potential severe depression (PHQ>=15) | 1 [0.32, 3.17] | 1.00 | ·· | 2.23 [0.78, 6.4] | 0.14 | ·· |
| **Having any unmet health need** | 0.52 [0.25, 1.08] | 0.08 | ·· | 0.56 [0.31, 1.02] | **0.06** | ·· |
| **Health expenditure per capita, difference from baseline** | 0.43 [-1.08, 1.95] † | 0.57 | 0.06 [-0.16, 0.29] | 0.69 [-0.30, 1.68] † | 0.18 | 0.13 [-0.06, 0.33] |
| **Second follow-up (16 months after the intervention)** | | | | | | |
| **Having any illness or injury** | 1.17 [0.75, 1.81] | 0.49 | ·· | 1.17 [0.71, 1.93] | 0.53 | ·· |
| **Respiratory conditions** | 1.08 [0.58, 1.98] | 0.82 | ·· | 0.78 [0.43, 1.42] | 0.41 | ·· |
| **Gastrointestinal conditions** | 1.03 [0.36, 2.99] | 0.95 | ·· | 1.09 [0.34, 3.45] | 0.89 | ·· |
| **Malaria** | 1.34 [0.76, 2.36] | 0.31 | ·· | 1.55 [0.82, 2.92] | 0.17 | ·· |
| **Chronic conditions** | 0.89 [0.42, 1.86] | 0.75 | ·· | 0.83 [0.36, 1.91] | 0.66 | ·· |
| **Pain-related conditions** | 1.47 [0.85, 2.55] | 0.17 | ·· | 1.52 [0.8, 2.89] | 0.20 | ·· |
| **Injury** | 1.02 [0.36, 2.9] | 0.97 | ·· | 0.75 [0.27, 2.07] | 0.57 | ·· |
| **Mental well-being** |  |  | ·· |  |  | ·· |
| Potential moderate depression (PHQ>=10) | 1.05 [0.62, 1.75] | 0.87 | ·· | 0.99 [0.54, 1.79] | 0.96 | ·· |
| Potential severe depression (PHQ>=15) | 0.65 [0.3, 1.41] | 0.27 | ·· | 0.6 [0.26, 1.4] | 0.24 | ·· |
| **Having any unmet health need** | 0.6 [0.32, 1.1] | 0.10 | ·· | 0.52 [0.26, 1.03] | 0.06 | ·· |
| **Having any unmet assistive products or services** | 0.96 [0.58, 1.57] | 0.86 | ·· | 1.02 [0.61, 1.72] | 0.93 | ·· |
| Mobility Aids | 0.72 [0.39, 1.31] | 0.28 | ·· | 0.71 [0.38, 1.3] | 0.27 | ·· |
| Glasses or contact lenses | 0.93 [0.56, 1.54] | 0.77 | ·· | 0.96 [0.55, 1.66] | 0.88 | ·· |
| Other sensory and communications tools | 1.07 [0.29, 4] | 0.92 | ·· | 0.89 [0.23, 3.38] | 0.86 | ·· |
| Others | 0.87 [0.34, 2.21] | 0.77 | ·· | 0.83 [0.32, 2.16] | 0.70 | ·· |
| **Health expenditure per capita, difference from baseline** | 0.29 [-1.39, 1.99] † | 0.73 | 0.04 [-0.2, 0.28] | 0.81 [-0.55, 2.17] † | 0.25 | 0.15 [-0.11, 0.42] |

**Sup Table 5. Health effects of the Disability-inclusive graduation programme on males with disabilities.** Intervention effects were estimated using two statistical approaches: (1) generalized estimating equations reporting odds ratios with 95% confidence intervals (CIs) for binary outcomes, and (2) linear mixed-effects regression reporting mean differences and standardized effect sizes with 95% CIs for continuous outcomes. The minimally-adjusted model included treatment status (fixed effect) and cluster/branch (random intercepts). Fully adjusted models additionally controlled for imbalanced variables (p<0.10): for first follow-up, including marital status and age of project participants, and symptom of anxiety of index person; for second follow-up including marital status and age of project participants, and household per capita income. †, result was reported as mean difference and its 95% CI.

| **Outcomes** | **Minimally adjusted analysis** | | **Effect size (95% CI)** | **Fully adjusted analysis** | | **Effect size (95% CI)** |
| --- | --- | --- | --- | --- | --- | --- |
|  | **Mean difference or odds ratio (95% CI)** | **p value** |  | **Mean difference or odds ratio (95% CI)** | **p value** |  |
| **First follow-up (0 month after the intervention)** | | | | | | |
| **Having any illness or injury** | 0.92 [0.56, 1.51] | 0.75 | ·· | 1.14 [0.68, 1.94] | 0.62 | ·· |
| **Respiratory conditions** | 0.55 [0.21, 1.45] | 0.23 | ·· | 0.67 [0.26, 1.76] | 0.42 | ·· |
| **Gastrointestinal conditions** | 1.99 [0.34, 11.52] | 0.44 | ·· | 1.99 [0.4, 9.91] | 0.40 | ·· |
| **Malaria** | 0.78 [0.39, 1.55] | 0.47 | ·· | 1.02 [0.48, 2.19] | 0.95 | ·· |
| **Chronic conditions** | 2.15 [0.96, 4.81] | 0.06 | ·· | 2.06 [0.91, 4.64] | 0.08 | ·· |
| **Pain-related conditions** | 0.96 [0.45, 2.07] | 0.92 | ·· | 1.15 [0.52, 2.56] | 0.72 | ·· |
| **Injury** | 0.77 [0.26, 2.27] | 0.64 | ·· | 0.7 [0.21, 2.37] | 0.57 | ·· |
| **Mental well-being** |  |  | ·· |  |  | ·· |
| Potential moderate depression (PHQ>=10) | 1.04 [0.57, 1.91] | 0.90 | ·· | 1.01 [0.51, 2.01] | 0.98 | ·· |
| Potential severe depression (PHQ>=15) | 3.26 [0.63, 16.72] | 0.16 | ·· | 2.86 [0.58, 14.12] | 0.20 | ·· |
| **Having any unmet health need** | 0.8 [0.33, 1.92] | 0.61 | ·· | 0.93 [0.38, 2.31] | 0.88 | ·· |
| **Health expenditure per capita, difference from baseline** | -0.27 [-2.47, 1.91] † | 0.81 | -0.03 [-0.31, 0.24] | -0.08 [-1.66, 1.49] † | 0.92 | -0.02 [-0.32, 0.29] |
| **Second follow-up (16 months after the intervention)** | | | | | | |
| **Having any illness or injury** | 0.97 [0.6, 1.55] | 0.90 | ·· | 0.97 [0.58, 1.64] | 0.92 | ·· |
| **Respiratory conditions** | 0.77 [0.34, 1.71] | 0.51 | ·· | 0.74 [0.31, 1.79] | 0.51 | ·· |
| **Gastrointestinal conditions** | 4.03 [0.8, 20.3] | 0.09 | ·· | 2.91 [0.58, 14.62] | 0.19 | ·· |
| **Malaria** | 1.49 [0.85, 2.63] | 0.16 | ·· | 1.46 [0.77, 2.76] | 0.25 | ·· |
| **Chronic conditions** | 0.86 [0.36, 2.04] | 0.73 | ·· | 0.86 [0.38, 1.94] | 0.71 | ·· |
| **Pain-related conditions** | 0.6 [0.33, 1.09] | 0.09 | ·· | 0.68 [0.36, 1.27] | 0.22 | ·· |
| **Injury** | 1.68 [0.51, 5.5] | 0.39 | ·· | 27.13 [11.84, 62.13] | < 0.001 | ·· |
| **Mental well-being** |  |  | ·· |  |  | ·· |
| Potential moderate depression (PHQ>=10) | 1.09 [0.61, 1.95] | 0.76 | ·· | 1.13 [0.59, 2.16] | 0.71 | ·· |
| Potential severe depression (PHQ>=15) | 1.44 [0.57, 3.63] | 0.44 | ·· | 1.45 [0.57, 3.72] | 0.44 | ·· |
| **Having any unmet health need** | 0.38 [0.16, 0.87] | **0.02** | ·· | 0.34 [0.14, 0.82] | **0.02** | ·· |
| **Having any unmet assistive products or services** | 0.83 [0.51, 1.34] | 0.45 | ·· | 0.77 [0.45, 1.3] | 0.32 | ·· |
| Mobility Aids | 0.68 [0.35, 1.34] | 0.27 | ·· | 0.65 [0.31, 1.34] | 0.24 | ·· |
| Glasses or contact lenses | 0.65 [0.39, 1.1] | 0.11 | ·· | 0.62 [0.34, 1.13] | 0.12 | ·· |
| Other sensory and communications tools | 0.04 [0.02, 0.12] | <0.001 | ·· | 0.04 [0.01, 0.1] | <0.001 | ·· |
| Others | 1.52 [0.58, 3.93] | 0.39 | ·· | 1.21 [0.46, 3.17] | 0.70 | ·· |
| **Health expenditure per capita, difference from baseline** | 1.28 [-0.83, 3.39] † | 0.23 | 0.17 [-0.11, 0.44] | 1.03 [-0.60, 2.69] † | 0.22 | 0.19 [-0.12, 0.5] |
