# Supplementary material for: Evaluation of health impacts of a disability-inclusive graduation programme among ultra-poor people with disabilities in Uganda: secondary analysis of a cluster randomized trial
Source: eClinicalMedicine. 2025 Jul 3;85:103318. doi: 10.1016/j.eclinm.2025.103318 (PMC12270699; doi:10.1016/j.eclinm.2025.103318)
Supplement: Protocol [file mmc2.pdf]

Kipchumba *et al. Trials* (2024) 25:206
https://doi.org/10.1186/s13063-024-08040-w

Trials

STUDY PROTOCOL

Open Access

Evaluation of a disability-inclusive ultra-poor graduation programme in Uganda: study protocol for a cluster-randomised controlled trial with process evaluation

Elijah Kipchumba[1,2†], Calum Davey[3,4†], Sarah Marks[3], Anthony Mugeere[5], Shanquan Chen[3], Lena Morgon Banks[3], Kazi Eliza Islam[6], Tom Shakespeare[3], Hannah Kuper[3*†] and Munshi Sulaiman[7†]

Abstract

Background There is little evidence on how to support ultra-poor people with disabilities to adopt sustainable livelihoods. The Disability-Inclusive Graduation (DIG) programme targets ultra-poor people with disabilities and/or women living in rural Uganda. The programme is an adaptation of an ultra-poor graduation model that has been shown to be effective in many contexts but not evaluated for people with disabilities.

Methods The DIG programme works with project participants over a period of 18 months. Participants receive unconditional cash transfers for 6 months, training, access to savings-and-loans groups, and a capital asset that forms the basis of their new livelihood. The programme is also adapted to address specific barriers that people with disabilities face. Eligible households are clustered by geographical proximity in order to deliver the intervention. Eligibility is based on household screening to identify the 'ultra-poor' based on proxy means testing—both households with and without people with disabilities are included in the programme. Clusters are randomly selected prior to implementation, resulting in 96 intervention and 89 control clusters. The primary outcome of the trial is per-capita household consumption. Before the start of the intervention, a baseline household survey is conducted (November 2020) among project participants and those not offered the programme, a similar endline survey is conducted with participants with disabilities at the end of programme implementation in July 2022, and a second endline survey for all participants in October 2023. These activities are complemented by a process evaluation to understand DIG programme implementation, mechanisms, and context using complementary qualitative and quantitative methods. Ethical approval for the research has been received from Mildmay Uganda Research Ethics Committee and London School of Hygiene and Tropical Medicine.

†Elijah Kipchumba, Calum Davey, Hannah Kuper, and Munshi Sulaiman contributed equally to this work.

†Calum Davey is joint first author.

†Hannah Kuper is joint last author.

*Correspondence:
Hannah Kuper
hannah.kuper@lshtm.ac.uk
Full list of author information is available at the end of the article

© The Author(s) 2024. **Open Access** This article is licensed under a Creative Commons Attribution 4.0 International License, which permits use, sharing, adaptation, distribution and reproduction in any medium or format, as long as you give appropriate credit to the original author(s) and the source, provide a link to the Creative Commons licence, and indicate if changes were made. The images or other third party material in this article are included in the article's Creative Commons licence, unless indicated otherwise in a credit line to the material. If material is not included in the article's Creative Commons licence and your intended use is not permitted by statutory regulation or exceeds the permitted use, you will need to obtain permission directly from the copyright holder. To view a copy of this licence, visit http://creativecommons.org/licenses/by/4.0/. The Creative Commons Public Domain Dedication waiver (http://creativecommons.org/publicdomain/zero/1.0/) applies to the data made available in this article, unless otherwise stated in a credit line to the data.

Discussion DIG is a promising intervention to evaluate for people with disabilities, adapted to be disability inclusive across programme components through extensive consultations and collaboration, and has proven efficacy at reducing poverty in other marginalised groups. However, evaluating a well-evidenced intervention among a new target group poses ethical considerations.

Trial registration Registry for International Development Impact Evaluations, RIDIE-STUDY-ID-626008898983a (20/04/22). ISRCTN registry, ISRCTN78592382. Retrospectively registered on 17/08/2023.

Keywords Disability, Income generation, Financial support, Social protection, Randomised controlled trial

Administrative information

Note: the numbers in curly brackets in this protocol refer to SPIRIT checklist item numbers. The order of the items has been modified to group similar items (see http://www.equator-network.org/reporting-guidelines/spirit-2013-statement-defining-standard-protocol-items-for-clinical-trials/).

Title {1	Evaluation of a disability-inclusive ultra-poor graduation programme in Uganda: study protocol for a cluster-randomised controlled trial with process evaluation
Trial registration {2a and 2b}.	Registry for International Development Impact Evaluations, RIDIE-STUDY-ID-626008898983a on (20/04/22). ISRCTN registry, ISRCTN 78592382. Retrospectively registered on 17/08/2023, https://www.isrctn.com/ISRCTN78592382.
Protocol version {3}	Version 2 (24/08/2020)
Funding {4}	The study is funded by the United Kingdom Foreign, Commonwealth and Development Office (FCDO) under the Programme for Evidence to Inform Disability Action (PENDA) (IATI Identifier: GB-EDU-133903-PENDA). Hannah Kuper is funded by a National Institute for Health and Care Research (NIHR) Global Research Professorship. Intervention implementation is conducted under a separate project led by BRAC Uganda, in partnership with Humanity and Inclusion (HI) and the National Union of Women with Disability in Uganda (NUWODU), with funding from FCDO under Inclusive Futures, Comic Relief and National Lottery.
Author details {5a}	Elijah Kipchumba[1,2]* & Calum Davey[3,4]* (joint first), Sarah Marks[3], Anthony Mugeere[5], Shanquan Chen[3], Lena Morgon Banks[3], Kazi Eliza Islam[6], Tom Shakespeare[3], Hannah Kuper[3]✉* & Munshi Sulaiman[7]* (joint last)<br>*These authors contributed equally to this work<br>✉Corresponding author<br>[1]Independent Evaluation and Research Cell (IERC), BRAC International, Kampala, Uganda; [2]Trinity College Dublin, Dublin, Ireland; [3]International Centre for Evidence in Disability, London School of Hygiene & Tropical Medicine, London, United Kingdom; [4] Centre for Evaluation, London School of Hygiene & Tropical Medicine, London, United Kingdom; [5]Department of Sociology and Anthropology, Makerere University, Kampala, Uganda; [6]BRAC International, Dhaka, Bangladesh; [7]BRAC Institute of Governance and Development, BRAC University, Dhaka, Bangladesh.
Name and contact information for the trial sponsor {5b}	BRAC Uganda<br>Plot 880, Heritage Road, Nsambya<br>P.O: Box 31817 (Clock Tower), Kampala, Uganda<br>T: + 256 (0) 714 274201, + 256 (0) 700861747<br>E: bracuganda@brac.net
Role of sponsor {5c}	Study sponsor, BRAC Uganda, is involved in the study design; collection, management, analysis, and interpretation of data; and writing of the report. Ultimate authority regarding study design and decision to submit the report for publication lies with LSHTM. The funders are not involved in any decision making with regard to the study design, implementation, analysis or publications.

Introduction

Background and rationale {6a}

The first Sustainable Development Goal (SDG) is to 'End poverty in all its forms everywhere', and this ambition has gained further urgency in the wake of the economic devastation caused by the Coronavirus Disease-2019 (COVID-19) pandemic. Social protection, often through cash transfers, has become a dominant mode for poverty reduction [1]. While an important policy tool, the size of the transfers can be too small to be transformative, leaving many recipients still in poverty [2, 3]. Recipients are rarely provided with the skills, assets, linkages with other services, and social networks needed to tackle the underlying drivers of poverty, making it difficult to break the poverty cycle [4].

As a result of these concerns, the non-governmental organisation (NGO) BRAC has developed an 'Ultra-Poor Graduation Programme' (UPG programme) that aims to help extremely poor people move out of poverty. This programme combines support for immediate needs with longer-term investments in skills training, asset transfers, enterprise development, saving, and planning. The UPG programme is characterised by a 'graduation' model that aims to lift poor households from extreme poverty into long-term sustainable livelihoods living in less-poor conditions.

BRAC's UPG programme has been delivered in several contexts around the world [1–3]. This includes a scaled programme in Bangladesh (2002–present), as well as pilot projects in Afghanistan (2010–2013), Pakistan (2010–2015), South Sudan (2013–2015), and Uganda (2016–Present). Randomised controlled trials (RCTs) of the graduation programme have been undertaken in

Bangladesh, Ethiopia, Ghana, Honduras, India, Pakistan, and Peru [1]. The trials investigated effects on multiple primary outcomes for each of the following domains: expenditure; food security; assets; finance; time use; income; physical health; mental health; political involvement; and women's decision making. Overall, there was evidence that the programme improved outcomes, although not physical health or women's decision making [1].

There are more than 800 million people with disabilities living in low- and middle-income countries that could benefit from the UPG programme [5]. People with disabilities are on average poorer than their peers without disabilities [5, 6]. Meaningful inclusion of people with disabilities in poverty reduction programmes is challenging because of multi-level barriers. These include negative attitudes and low expectations held by programme staff, family members and people with disabilities themselves, poor physical or informational accessibility, lack of access to needed accommodations, and failure of programmes to consider disability-related extra costs [3, 7]. Previous impact evaluations of the UPG programme have not included people with disabilities and there is consequently a lack of evidence regarding whether the graduation model can be adapted to their needs and the impacts of such an adapted model—important questions for development policy and programming.

BRAC Uganda has been funded to adapt the UPG programme for households of people with disabilities in Uganda with funding from the United Kingdom's Foreign Commonwealth and Development Office (UK FCDO), as part of Inclusive Futures, Comic Relief, and the National Lottery. The project is referred to as the 'disability-inclusive graduation' (DIG) programme and is implemented in partnership with Humanity and Inclusion (HI) and the National Union of Women with Disability in Uganda (NUWODU), a national Organization of Persons with Disabilities (OPD).

Implementation of the DIG programme in Uganda provides an opportunity to determine whether it can have a positive effect on people with disabilities' livelihoods and well-being. The FCDO-funded Programme for Evidence to Inform Disability Action (PENDA), led by London School of Hygiene and Tropical Medicine (LSHTM), aims to fill the evidence gap in disability and development. PENDA will evaluate the DIG programme, in partnership with BRAC Institute of Governance and Development (BIGD) and its affiliated Independent Evaluation and Research Cell (IERC) in BRAC International, which works independently of BRAC Uganda. It is hoped that the outcome of the evaluation will inform governments and other NGOs seeking to deliver similar programmes for people with disabilities.

Objectives {7}

The aim of the impact evaluation is to estimate the effect of the DIG Programme on the livelihoods and well-being of people with disabilities and their families in Uganda. Consequently, the primary objective is to estimate the effect of the DIG programme on per-capita expenditure, livelihood, and social participation of people with disabilities and their families. The secondary objective of the impact evaluation is to estimate the differential effect of the DIG programme on per-capita expenditure, livelihood, and social participation of people with disabilities and their families, compared to the effect among people without disabilities. The study is complemented by a process evaluation. The objectives of the process evaluation, in accordance with MRC guidance for process evaluations of complex interventions [8], are to describe the intervention implementation as delivered, test evaluation hypotheses, and generate theoretical learning to inform future intervention designs.

Trial design {8}

The overall effect of the intervention is estimated using a two-arm, parallel group, longitudinal, pair-matched cluster-randomised controlled superiority trial design. Clusters are defined as villages with 10–75 eligible households, within which households act as the observational unit (see 'Eligibility criteria {10}' section for further details).

The DIG programme has identified more than 11,000 eligible households, but only has funding to reach 2700 households under the programme. Randomisation is therefore an equitable way to select households for inclusion. It is also not yet clear that the intervention is effective for people with disabilities and their families. Cluster randomisation was chosen in part because of the nature of the DIG programme, which includes components delivered at the village level.

After random allocation of villages into intervention and control arms, but prior to implementation of the intervention, a baseline survey at household level is conducted (November 2020). The DIG programme is then implemented for 18 months from December 2020 to June 2022. Two endline household surveys are undertaken—the first in July 2022 for participants with a disability to determine the effects of DIG at programme end (endline 1); and a second one in October 2023 for all participants (endline 2), inclusive of participants with disabilities, to assess the longer-term differential effects of the DIG programme between households with and without disabilities.

This work is complemented by a process evaluation to further understand implementation of the DIG programme. The process evaluation is guided by theoretical

considerations, such as What underlying theory are we testing with this intervention? What is the theory underlying the design of the graduation programme? And what is the theory underlying the adaptations to make them inclusive? These considerations ground the process evaluation in the relevant wider literature, and support generalisability to future intervention designs that draw on similar theories. To facilitate consideration of theory, an unambiguous articulation of project theory as supposed by the delivery team is developed iteratively in the form of a directed acyclic graph (DAG), based on the theory of change and using set rules for specifying how change is expected to happen.

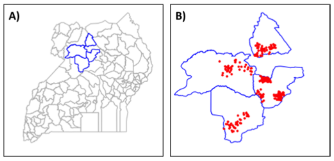

Fig. 1 Study districts (**A**) and villages (**B**). Study district boundaries in blue, while study villages are shown as red dots

Methods: participants, interventions and outcomes

Study setting {9}

Kiryandongo, Gulu, Nwoya, and Oyam districts in Uganda are the selected sites for DIG programme implementation. These districts are characterised by higher than national average levels of poverty, social deprivation, and prevalence of disability. A map of programme locations is shown in Fig. 1.

Eligibility criteria {10}

For the purposes of intervention delivery and evaluation, clusters are villages with 10–75 eligible households. This cluster size is necessary for the Village Savings and Loans Associations (VSLAs) and Village Poverty Reduction Committees (VPRCs) (described further under *Intervention description*). To create a cluster, either nearby small villages are grouped or larger villages split to create a manageable cluster, that is, having between 10 and 75 eligible households. For small villages, the geographic distance from its centroid was calculated to the centroid of its adjacent villages, using eligible household's GPS coordinates. The village concerned was then grouped with adjacent villages having the shortest distance. To split larger villages, we relied on *k*-means clustering to iteratively group neighbouring households, using their GPS coordinates, within a village into clusters. From the 156 villages identified near the BRAC Uganda branch offices in the target districts as potential sites for the DIG programme, 185 of these artificial clusters were created, each with at least 10 eligible households.

Identification of eligible households is based on household-level data collected by the implementing partners (BRAC, HI, and NUWODU). Households are eligible if they meet at least three out of five criterions, as this was felt to be an adequate threshold and would also reflect variations in household experiences of poverty. That is, (1) having a person with disability, (2) being a female-headed household or dependent on earnings from a female member of the household, (3) having children who are out of school, (4) poor housing conditions (floor, roof, and wall), and (5) low productive asset endowment. The Washington Group short set of questions is used to identify households with people with disabilities, based on those that report 'a lot' of difficulty on at least one dimension of the six-question set (i.e. walking, seeing, hearing, communicating, understanding and self-care) [8]. Ultra-poor households without a person with disabilities are still eligible for inclusion in the DIG programme in order to ensure there are enough households within each cluster for village-level interventions to operate.

The unit of participation in the programme is the household; however, a single individual within each household is the 'project participant' who is the main recipient of the training and enterprise. This person is expected to take responsibility for managing the enterprise and participate in the programme activities, such as training on enterprise management or savings group meetings. At least 15% of the project participants should be people with disabilities, in line with the proportion of Ugandans living with a disability [9]. Women are also prioritised as project participants, in line with previous UPG programme implementation, and to promote gender equity within DIG for participants with and without disabilities. Children (i.e. people aged below 18 years) are not eligible to be project participants within the DIG programme.

BRAC and collaborators have developed a suite of locally appropriate enterprise options that are accessible to people with disabilities. However, for households where a person with a disability is not able to manage the available enterprise options, such as a person with severe cognitive impairment, the primary caregiver for that person may act as the participant instead.

Interventions

Explanation for the choice of comparators {6b}

Participants in the control arm do not receive the DIG programme. Control arm participants identified as having disabilities are counselled on how and where to seek health and/or rehabilitation services that they may require. Additionally, they are provided with information on all the social protection programmes to which they

may be eligible, and how to make an application. This is a suitable comparator to the intervention, as it enables the study to establish if the DIG programme has any additional impact as compared to currently available services.

Intervention description {11a}

The four main graduation programme components of DIG are:

(1) Livelihood: The livelihood component should lead to improved enterprise management skills, asset accumulation or diversification, and increased income, through receipt of assets (e.g. livestock), technical training, and individual-level support for income generation. Assets are chosen based on local market opportunities and the skills and capabilities of the recipients.
(2) Social protection: Social protection should increase access to health services, social safety nets, and support mechanisms and consequently improve household food intake and dietary diversity and improve health. Intervention activities under this component includes unconditional cash transfers for 6 months; healthcare subsidy and rehabilitation, physiotherapy, and psychosocial support; activities to support beneficiaries to overcome access barriers to government and NGO social entitlements (both disability-specific and general); and support services, including health, education, and social protection.
(3) Financial inclusion: Financial inclusion should result in improved financial management skills and increased savings, developing ability and confidence to access financial services, cope with shocks, and invest in productive assets. Activities encompass: financial literacy training; village savings and loans association (VSLAs) formation; and on-going coaching. Training materials are adapted by HI and NUWODU to ensure that the VSLAs do not exclude people with disabilities.
(4) Social empowerment: Social empowerment should result in better social integration within households and communities and improve participant confidence and aspirations. DIG provides home coaching for individual counselling and life-skills, individual empowerment plans (supported by HI), and the formation of inclusive Village Poverty Reduction Committees (VPRCs). VPRCs act as local governance structures with government/community leadership representation. The presence of these, and the VSLAs, means that while principally a household-level programme, the programme also has important village-level components.

Additional disability-specific components are intended to reduce barriers and enable inclusive participation in the poverty graduation programme. These disability-specific components include (1) access to occupational, physical and psychosocial therapy, and referrals through local technical staff hired by the project; (2) bi-monthly home visits provide inclusive life-skills training, coaching, and emotional support; (3) attitudinal barriers among project participants, project staff, BRAC Uganda staff, and key external stakeholders are addressed through sensitivity training provided by NUWODU and HI. The sensitivity training aims to improve disability inclusion at the organisational level by conducting a disability inclusion self-assessment of BRAC Uganda, led by NUWODU and HI, to develop a disability inclusion plan, and monitor its implementation—including changes at programme, management, human resources, and policy levels; (4) The programme supports advocacy, including sensitising village leaders on disability inclusion through NUWODU's District Women's Associations. DIG aims to shift norms and behaviours at the community level by bringing together local OPD leaders, local and religious leaders in VPRCs to advocate for the empowerment of people with disabilities. DIG also delivers disability-awareness training to civil-society organisations.

A Theory of Change (ToC) describing the DIG programme is shown in Fig. 2.

Criteria for discontinuing or modifying allocated interventions {11b}

Project participants and households can choose to discontinue participating in the intervention or study at any time with no obligation to give a reason for withdrawal and without prejudice to future engagement in the DIG programme or other BRAC projects. No modification of allocated interventions will be conducted.

Strategies to improve adherence to interventions {11c}

No specific strategies to improve adherence to interventions. However, a compliance survey undertaken by BRAC Uganda mid-way through DIG programme implementation (July 2021) is used to determine engagement with the intervention components among project participants.

Relevant concomitant care permitted or prohibited during the trial {11d}

No restrictions on concomitant care.

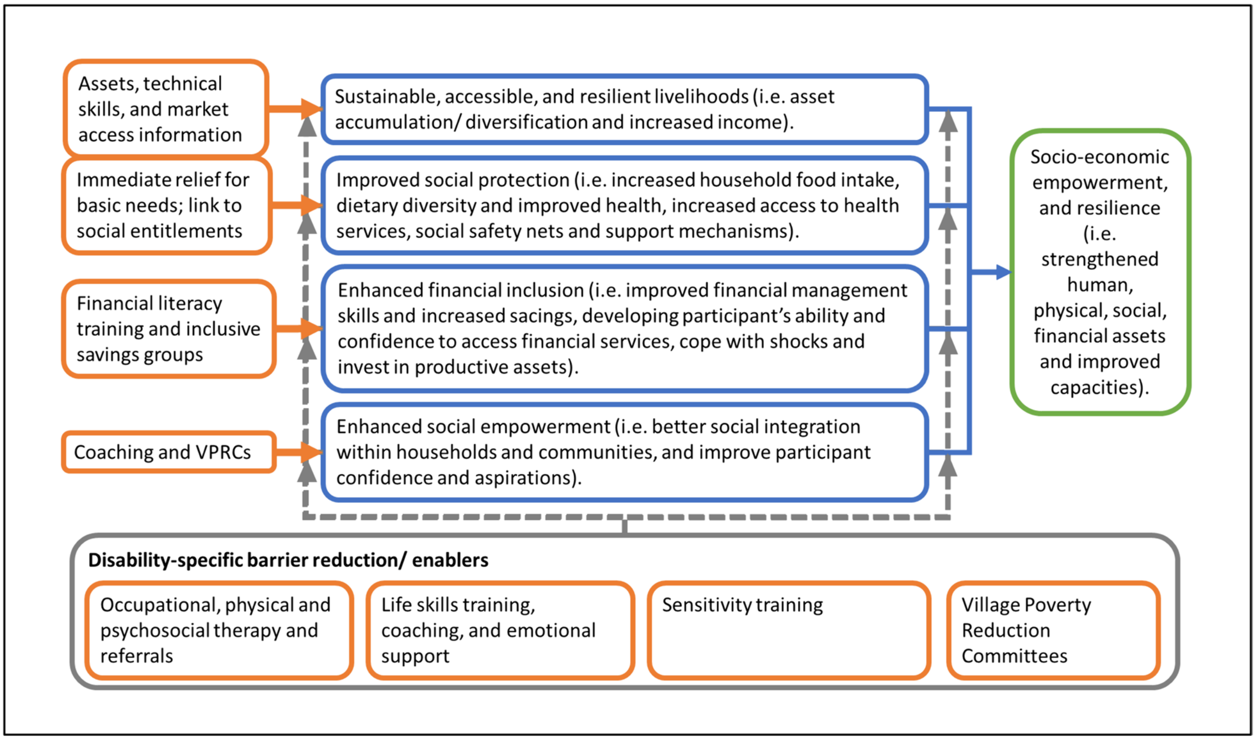

Fig. 2 Theory of change for the DIG programme

Provisions for post-trial care {30}

No specific provisions for post-trial care.

Outcomes {12}

The primary outcome is per-capita annual household expenditure, which is arrived at by dividing the total annual household expenditure by the number of individuals that habitually reside in the household. Expenditures covered include food items, non-food household items (e.g. electricity, utilities), healthcare, and education, collected in Ugandan Shillings. Amounts are adjusted for inflation and expressed as constant 2017 purchasing power parity (PPP) United States Dollars. Household expenditure has a closer link with household well-being and is often used to evaluate transfer programmes. Household poverty status will also be derived, whether per-capita consumption expenditure is below US$1.90 per day and the household poverty gap, the shortfall in per-capita consumption expenditure from the poverty line—US$1.90 per day. Further secondary outcomes measure anticipated changes based on the theory of change. These are annual household income from agricultural and non-agricultural sources in the last 1 year preceding the survey (household level); participation in livelihood activities by project participants; participation in social activities by project participants in the last 1 week preceding the survey; and the health and well-being of project participants. For the process evaluation, outcomes include the programme's fidelity, reach, and dose; mechanisms of impact (e.g. mediators); and context-dependencies (e.g. moderators).

Participant timeline {13}

Table 1 shows the participant timeline.

Sample size {14}

The DIG programme plans to reach 2700 households, of whom at least 15% of the project participants are people with disabilities, in line with the proportion of Ugandans living with a disability [9]. Thus, a minimum of 405 project participants are to be people with disabilities.

The sample size calculations are based on the effect of the intervention on the primary outcome among people with disabilities. The number of programme recipients, and consequently the size of the study, is fixed by the implementation partners' funding. These calculations

determine whether the study has sufficient precision to estimate the effect on per-capita expenditure in households with people with disabilities as the project participant.

Available data from the screening and verification visits informed the estimated total number of clusters that would be needed to reach 5400 eligible households. During the screening and verification process, BRAC Uganda found that approximately 25% of project participants were people with disabilities, and as such it was expected that the trial would include 675 people with disabilities in each arm. The distribution of eligible households per village was used to simulate the trial, 185 villages sampled with replacement from the verified villages gave approximately 5400 research participants in a simulated trial. A value of 0.8 was used from a recent evaluation of the graduation programme in Ghana for the standard deviation of the distribution of the normalised per-capita expenditure at baseline [10]. Since the degree of clustering in the outcome was unknown, the intraclass correlation coefficient (ICC) was allowed to vary between 0.05 and 0.3. The estimated between-village variation in mean level of the outcome at baseline and individual-level outcomes were drawn from the village-level means with a constant within-village variance. The endline results were generated using the simulated baseline data and a covariance matrix with a pre-post correlation of either 0.4, 0.6, or 0.8. The 185 clusters were pair-matched by the number of people with disabilities and a constant intervention effect was applied to the clusters allocated to the intervention arm. The intervention effects ranged from a difference between the intervention and control arms in the mean per-capita expenditure of zero standard deviations of the baseline per-capita expenditure, to 0.2 standard deviations, which is as high as the largest effects on per-capita expenditure observed in previous trials [1]. Multi-level linear regression was used to estimate the effects, controlling for baseline per-capita expenditure, accounting for clustering with a village-level random effect, and for the randomised design with fixed-effect for the pairs.

The results from the simulated analyses are shown in Fig. 3. The darker CIs correspond to scenarios with higher ICCs. Estimates were precise when the pre-post correlation was high (right-hand panel), and sufficiently precise to exclude the null with a low correlation and true effect of a difference between the arms of 0.1 standard deviation of per-capita expenditure at baseline (left-hand panel). Under conservative assumptions, we would reject the null hypothesis at conventional levels ($p < 0.05$) if the true effect of the intervention on per-capita expenditure was at least 0.1 standard deviations of the baseline distribution. This effect size is similar in magnitude to the overall effect on per-capita expenditure found in trials of the original (non-inclusive) version of the programme [1]; therefore, this trial would have sufficient power to detect an effect if the adaptations of the intervention for people with disabilities worked such that the effect was as strong as it had been for people without disabilities.

How big is 0.1 standard deviations in per-capita expenditure in real terms? Pooled analysis of studies in six countries shows that the 0.1 standard deviation effect on per-capita expenditure was equivalent to US$5 per-capita per month (adjusted for purchasing power parity [1]). In households with on average six members, this would amount to US$27 increased expenditure per month per household. Many ultra-poor families live on less than $US0.80 per person per day, and this would constitute an 18% increase in per-capita expenditure. In contrast, where available, disability allowances in Uganda amount to around $5 per person with disability per month [7]. Therefore, an effect of 0.1 standard deviations would be a substantial increase compared to other benefits.

An effect of this size would also have policy implications since it would likely mean that the programme was cost effective. Aside from Honduras (where there was a negative effect on expenditure), in previous studies the UPG programme had cost-effectiveness ratios between 133% in Ghana to 433% in India, based on the improvement in per-capita expenditure alone [1]. Such a result for people with disabilities would be important not only for the families involved, but also for motivating investment in an under-served population.

Recruitment {15}

The implementing partners (BRAC, HI, and NUWODU) are responsible for selection of households to take part in the programme. Since the graduation model entails a large investment in participating households, accurate targeting of beneficiary households is important to avoid waste [11]. Selection into the programme is based on household-level indicators, and achieved by a two-stage process:

> *Stage 1*: Selection of potentially eligible households. BRAC Uganda screens households in villages near to their branch offices in the target districts. Eligible households are identified based on the eligibility criteria, as earlier described.

Table 1 Participant timeline for the DIG programme's cluster randomised controlled trial

Visit	(1) Screening	(2) Verification	RANDOMISATION	(3) Baseline	DIG Programme Implementation			(4) Endline 1 (Participants with disabilities)	(5) Process Evaluation	(6) Endline 2 (All participants)
**Timepoint**	**July 2020**	**August 2020**		**November 2020**	**(December 2020-June 2022)**<br>1–6 months	6–12 months	12–18 months	**July 2022**	**January–February 2023**	**October 2023**
**Conducted by**	BRAC Uganda	BRAC Uganda		BIGD	BRAC Uganda	BRAC Uganda	BRAC Uganda	BIGD	Makerere University	BIGD
**Activities**	☐ Household survey to screen for eligibility	☐ Verification of ultra-poor households<br>☐ Identification of project participants		☐ Informed consent of respondents<br>☐ Household survey with female head of household and person with disabilities (if not female head of household)	☐ Training<br>☐ Asset transfer<br>☐ One-to-one follow-up and supervision<br>☐ Social development and health services support<br>☐ Expenditure support: a regular transfer of cash	☐ Training<br>☐ Further asset transfer<br>☐ One-to-one follow-up and supervision<br>☐ Group formation<br>☐ Village savings and loans groups<br>☐ Informal group discussions<br>☐ Social development and health services support<br>☐ Compliance survey via phone with female head of household in intervention arm (with informed consent)	☐ Training<br>☐ Individual follow-up<br>☐ Group discussion<br>☐ Confidence-building training<br>☐ Social development and health services support<br>☐ Formal weekly meetings<br>☐ Loan activities<br>☐ Savings activities<br>☐ Monthly group-level follow-up	☐ Informed consent of respondents<br>☐ Household survey with female head of household and person with disabilities (if not female head of household). Limited to just households with a person with disabilities	☐ Informed consent of respondents<br>☐ Semi-structured interviews with programme beneficiaries and key informants	☐ Informed consent of respondents<br>☐ Household survey with female head of household and person with disabilities (if not female head of household)

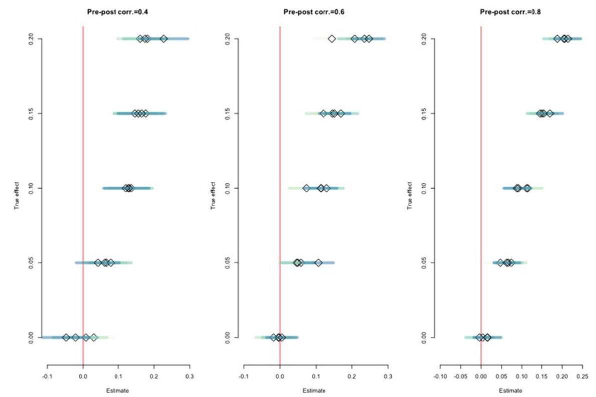

Fig. 3 Precision of estimates of effect using simulated data. Darker confidence intervals are from scenarios with higher levels of clustering

> *Stage 2*: Verification of eligibility. Verification is completed by the project manager of the DIG programme. The project manager confirms that households meet the eligibility criteria, as earlier described.

The unit of participation in the programme is the household; however, a single individual within each household is the 'project participant' who is the main recipient of the training and assets. This person is expected to take responsibility for managing the enterprise and participate in other activities, such as training on enterprise management or savings group meetings. Project participants are selected before randomisation. The field worker and the household members discuss who is best suited to manage the enterprise and take part in the training. When discussing with household members who should be the participant, the fieldworkers encourage people with disabilities and/or women—usually spouse to the household head—to be the participants where possible even if they are not the head of household.

Who will take informed consent? {26a}

For the evaluation, informed consent is obtained prior to each round of data collection. The informed consent process is conducted by the BIGD/IERC interviewer, trained in the informed consent process. Before taking informed consent, the interviewer provides hard copies and reads aloud the participant information sheet and consent forms to the participants. Consent and data collection procedures are adapted to support the participation of people with different impairments, such as sign language for participants with profound hearing impairments and knowledge of a standard sign language. Written informed consent is then obtained by means of participant-dated signature (or thumbprint) and dated signature of the person who presented and obtained the consent.

Additional consent provisions for collection and use of participant data and biological specimens {26b}

Participants provide their consent for their data to be uploaded into a public repository and for it to be used in ancillary studies during the informed consent process (see Supplemental file 1 for model consent form). No biological specimens will be collected.

Assignment of interventions: allocation

Sequence generation {16a}

Randomisation is stratified by BRAC office branch, to ensure sufficient programmatic support to the clusters in the intervention arm, and pair-matched based on the number of people with disabilities. For each branch, clusters are ranked by the number of project participants who are people with disabilities. Adjacent clusters in the ranked lists are paired. Each cluster in a pair is randomly allocated to each arm using random number allocation in using Stata (StataCorp. College Station, TX: StataCorp LLC).

Concealment mechanism {16b}

No concealment mechanism, clusters are randomly allocated using Stata (StataCorp. College Station, TX: StataCorp LLC).

Implementation {16c}

Assignment of clusters to intervention and control arms is led by the implementing partners (BRAC, HI, and NUWODU) with support from IERC and BIGD, in order to meet the criteria for the evaluation. Allocation is conducted at one time point prior to the baseline survey and implementation of the DIG programme. The programme team pre-identified the desired number of intervention beneficiary households that each of the eight BRAC branches could support based on the sample size of 2700 households. This number ranged from 320 to 420 households.[1] The number of eligible households, identified during the screening and verification process, within each cluster was calculated. Permutations of cluster allocation were then run in Stata, until the desired total number of 2700 households for the DIG programme was reached in the intervention arm. Remaining clusters were assigned to the control. In the end, 96 clusters were assigned to the intervention arm and the remaining 89 clusters to the control arm. Overall, 2898 households were assigned to the intervention arm while 2402 households were retained as control—the distribution of households by district and BRAC branch is shown in Table 2.

[1] Loro (320), Lacor (350), Kigumba (360), Gulu (370), Kamdini (380), Anaka (380), Goma (420) and Minakulu (420).

Table 2 Distribution of households in control and intervention arms by district and BRAC branch

District	BRAC Branch	Number of Households		
		Control Arm	Intervention Arm	Total
Gulu	Gulu	367	355	722
	Lacor	288	340	628
	Goma	280	406	686
Nwoya	Anaka	366	370	736
Oyam	Minakulu	324	402	726
	Kamdini	285	357	642
	Loro	263	311	574
Kiryandongo	Kigumba	229	357	586
Total		2402	2898	5300

Assignment of interventions: masking

Who will be masked {17a}

Post-intervention allocation, there is no masking as households and project participants in clusters assigned to intervention or control will be aware of their allocation given the nature of the intervention.

Procedure for unmasking if needed {17b}

Not applicable.

Data collection and management

Plans for assessment and collection of outcomes {18a}

Baseline data is collected for the impact evaluation (November 2020), before the DIG programme starts; after the DIG programme ends following 18 months of implementation, data is collected for the first endline from households with people with disabilities (July 2022), and for the second endline from all households and participants (October 2023). Data is collected for the baseline and endlines using questionnaires that have been piloted prior to use. Questionnaires are administered by experienced and trained data collectors, overseen by BIGD/IERC, to members of selected households in both the control and intervention arms (see Table 3 for questionnaire domains, full questionnaires available in Supplemental file 2). One questionnaire refers to features of the household and should be completed by the female head of household. Another questionnaire refers to the person with disabilities and should be completed by this person, if different to the female head of household. The female head of household survey includes a tool that has been adapted and verified for the Ugandan context to measure per-capita monthly expenditure at the household level. The survey for the person with disabilities collects disability-related information and was adapted from the World Health Organization's Model Disability Survey [12].

The process evaluation uses data from the impact evaluation in conjunction with other data sources. This includes programme monitoring data collected by BRAC Uganda at the branch and national level, such as records of asset transfer timings, number of household visits (and by whom), and other project activities. A compliance survey conducted by BRAC Uganda as part of their programme monitoring activities is conducted halfway through programme implementation (July 2021), this will inform the level of exposure to the intervention among project participants. This is complemented by questions in the endline surveys on exposure to intervention components. Qualitative interviews with the project staff (approximately 10) and project participants (approximately 30; 15 in each arm, 10 with disabilities and 5 without) are conducted at the end of the project to address domains such as fidelity, adaption, reach, mediators, unintended consequences, and context of programme implementation, and to further understand participant responses to the intervention. The endline surveys also

Table 3 Questionnaire domains

Female head of household	Person with disability
☐ Identification and household characteristics<br>☐ Household welfare and assets<br>☐ Household expenditure<br>☐ Household agriculture, livelihoods, and other income-generating activities<br>☐ Household loans and savings<br>☐ Household use of assistance<br>☐ Food security<br>☐ Vulnerability to shocks<br>☐ Health of household members	☐ Self-rated health / perceived well-being; self-stigma/negative attitudes<br>☐ Quality of life<br>☐ Household decision making<br>☐ Participation<br>☐ Attitudes & behaviour of others<br>☐ Environmental factors<br>☐ Personal assistance and assistive devices

provide data for the process evaluation regarding factors expected to be on the causal pathway, and contextual factors. Further information on the process evaluation domains can be found in Supplemental file 3.

Plans to promote participant retention and complete follow-up {18b}

Consent and data collection procedures are adapted to support the participation of people with different impairments (e.g. sign language for participants with profound hearing impairments and knowledge of a standard sign language). Interviews take 60–120 min per visit and could be tiring for participants. To compensate for this fatigue, participants are offered a small token (bar of soap or a kilogramme of sugar) at the end of the interview as an appreciation. Participants are given the opportunity to stop at any time if they become fatigued and would no longer like to continue participating.

Data management {19}

Quantitative data collection is conducted by IERC and BIGD, and qualitative data collection for the process evaluation done by Makerere University. Qualitative data collected through interviews with beneficiaries and key informants are audio recorded, then transcribed and translated into English. Audio recordings and transcripts are securely stored by the head of the qualitative evaluation team. Quantitative data, collected through the household surveys, is collected electronically using SurveyCTO (Dobility, Inc. Cambridge, MA, USA), where all data is password protected on devices and servers. Once BIGD have cleaned and ensured the data is anonymized, data is shared with LSHTM in the UK using a secure data transfer protocol, where it is securely stored on LSHTM servers.

Confidentiality {27}

All study staff undergo ethics training and sign a confidentiality agreement. The research team ensures that all research data collected are anonymised using unique identification numbers.

Plans for collection, laboratory evaluation and storage of biological specimens for genetic or molecular analysis in this trial/future use {33}

Not applicable, as no biological specimens collected.

Statistical methods

Statistical methods for primary and secondary outcomes {20a}

For basic description, we will report the mean and standard deviation (or median and interquartile range) for continuous variable and the number and percentage for categorical variables, by intervention status (yes or no), and by data wave (baseline vs follow-up). We will test the balance of the covariates by t-test (or Mann Whitney U test) for continuous variables and by chi-square test for any categorical variables. Covariates include socio-demographic factors (e.g., age, sex), as well as any other factors likely to affect the implementation of the intervention.

Data will be fitted with multi-level linear regression to test the effect of the intervention, with the difference of the primary or secondary outcomes between follow-up and baseline as the outcome, and intervention status as the exposure, controlling for potential imbalance in covariates at baseline. To account for the data structure, we will consider the random intercept and random slope for exposure at the level of district and pair indicators. To address multiplicity concerns the Bonferroni Correction or Bayesian modelling approaches will be applied. The model's adequacy will be assessed through residual plots, with log transformations of variables considered if residuals are not randomly distributed. All analysis will be conducted in Stata and R. We will report the estimation as coefficient for continuous outcome and OR for binary outcome, as well as their 95% confidence interval (CI). P value < 0.05 will the level of statistical significance.

Interim analyses {21b}

No interim analyses.

Methods for additional analyses (e.g. subgroup analyses) {20b}

Subgroup analyses will be conducted to ascertain the differential efficacy of the intervention. These analyses will include stratification by sex, disability status (whether the household has people with disabilities), and by whether the recipient of the intervention is the person with disabilities within the household. The objective of these subgroup analyses is to elucidate whether the intervention's effectiveness varies across these distinct demographic segments, thereby providing a deeper insight into its targeted impact.

For the process evaluation, we will combine data sources to determine fidelity, reach and dose, in order to describe the actual intervention as experienced by programme participants. To identify mechanisms of impact, we are working with the FCDO-funded POInT (process-outcome integration with theory) project led by LSHTM [13], to build a framework for jointly interpreting certain aspects of the qualitative and quantitative data gathered to make inferences about the causal pathways that are most important for this intervention to work (mediators). Similarly, for general theoretical learning, we will use qualitative data to learn about unexpected context

contingencies, and quantitative data to explore moderation by contextual factors.

Methods in analysis to handle protocol non-adherence and any statistical methods to handle missing data {20c}

The full analysis set (FAS) will be defined according to the intention-to-treat (ITT) principle. The FAS will consist of all randomised subjects analysed according to the study arm to which they were assigned at randomisation. Participants who withdraw consent for continued follow-up will be included in the analysis by modern imputation methods for missing data as sensitivity analysis. Where possible, reasons for withdrawal for each group will be reported and compared qualitatively.

Plans to give access to the full protocol, participant-level data, and statistical code {31c}

Twelve months after the end of the study, the anonymized survey data will be made available on LSHTM's Data Compass (datacompass.lshtm.ac.uk), for which explicit consent has been included in the consent form, alongside project documentation and a data user guide.

Oversight and monitoring

Composition of the coordinating centre and trial steering committee {5d}

BRAC Uganda is responsible for the intervention design, allocation, implementation, and programme monitoring. BIGD and IERC lead household survey data collection, and Makerere University leads qualitative data collection for the process evaluation. Evaluation design and analysis is led by LSHTM, in partnership with BIGD, IERC, and Makerere University. Monitoring data collected from implementing partners is shared with LSHTM for the purpose of the process evaluation.

Composition of the data monitoring committee, its role and reporting structure {21a}

As the evaluation is not blinded and the interventions pose a limited risk to participants, a data monitoring committee is not required.

Adverse event reporting and harms {22}

Delivery of interventions is monitored by BRAC Uganda. As the intervention is targeted at the worst-off households, many households within the intervention villages do not actually receive the intervention. It is possible that the targeting methods are imperfect or appear imperfect to members of the communities, and that there is resentment within the villages. This is not a feature of the random allocation but is a feature of the programme. Resentment may lead to perpetration of violence of theft, particularly targeting people with disabilities, which is monitored by BRAC Uganda through a feedback and complaint handling mechanism, and will inform the process evaluation.

Frequency and plans for auditing trial conduct {23}

Data is quality assured in the field by the data collection team supervisor and checked after submission by the data manager. Any discrepancies are followed up with the relevant data collector as required.

Plans for communicating important protocol amendments to relevant parties (e.g. trial participants, ethical committees) {25}

Any important modifications to the study protocol are agreed between BRAC Uganda, Makerere University, and LSHTM, and approved by LSHTM Ethics Committee and Mildmay Uganda Research Ethics Committee prior to implementation.

Dissemination plans {31a}

To disseminate findings from this research, we will write academic articles, present at conferences and publicise the results through our network of academic and non-academic partners.

Discussion

The DIG programme is an ambitious attempt to deliver an intervention that aims to sustainably change the levels of poverty among people with disabilities in rural Uganda. There is currently a lack of evidence on the impact of interventions designed to improve livelihoods among people with disabilities [14]. DIG is a promising intervention to evaluate, as it has been adapted to be disability inclusive across programme components through extensive consultations and collaboration with people with disabilities, OPDs, and other disability experts. Furthermore, it has proved effective at reducing poverty among other target groups in several different contexts [1].

This is a pragmatic trial [15], where the intervention is being delivered in 'real-life' conditions. Pragmatic trials delivered in 'real-life' conditions have advantages and disadvantages for informing policy. They are inherently realistic, so any evidence of effect is a good indication that a scaled-up programme could replicate the effects. However, by being so dependent and contingent on the context at all levels, generalising the effects (or lack thereof) to other settings—or even the same setting in the future [16]—can be challenging. We are addressing this disadvantage with a theory-driven

process evaluation that will identify key mediators and moderators of the processes that occurred and will combine this with other data to make recommendations on how interventions should be informed by this evaluation when delivered elsewhere.

Finally, pragmatic trials have challenges relating to the ethics of randomisation and data collection from people who are not directly receiving the intervention. Questions arise since the programme is not an intervention developed for the purposes of research about which there is 'equipoise', but an NGO-led programme that has been shown to be effective in other contexts. The decision to randomise was itself pragmatic; the funds were limited, and many more eligible people had been identified. More importantly for the cause of generating evidence for people with disabilities, while the intervention has been shown to be effective before it has not been for people with disabilities. Given the programme's emphasis on inclusion, it is difficult to design an intervention that would both allow all people without disabilities to get the intervention (because it has been previously shown to be effective) but not people with disabilities (because a trial is justified by the lack of evidence). Striking the balance between the interests of generating evidence for a marginalised group and rolling out a proven approach to poverty alleviation is a challenge for this and for future research.

Trial status

This manuscript is based on protocol version 2, 24 August 2020. Baseline data collection and recruitment was conducted in November 2020. The first endline evaluation was conducted in July 2022 and the second endline is planned for October 2023. It was not possible to submit the manuscript prior to participant recruitment as funding for the second endline was pending (now approved) and this would have influenced the study design.

Abbreviations

BIGD	BRAC Institute of Governance and Development
CI	Confidence interval
COVID-19	Corona virus disease 2019
DIG	Disability-inclusive graduation programme
FAS	Full Analysis Set
FCDO	Foreign, Commonwealth and Development Office
GPS	Global Positioning System
HI	Humanity and Inclusion
ICC	Intraclass correlation coefficient
IERC	Independent Evaluation and Research Cell
ITT	Intention-to-treat
LSHTM	London School of Hygiene and Tropical Medicine
MRC	Medical Research Council
NGO	Non-governmental organisation
NUWODU	National Union of Women with Disabilities of Uganda
OPD	Organization of persons with disabilities
PENDA	Programme for Evidence to Inform Disability Action
POInt	Process-outcome integration with theory
RCT	Randomised controlled trial
SDG	Sustainable development goals
ToC	Theory of change
UK	United Kingdom
US	United States
UPG	Ultra-poor graduation programme
VPRC	Village Poverty Reduction Committees
VSLA	Village Savings and Loans Associations

Supplementary Information

The online version contains supplementary material available at https://doi.org/10.1186/s13063-024-08040-w.

Supplementary Material 1.

Supplementary Material 2.

Supplementary Material 3.

Acknowledgements

Eliud Marangu, BRAC Uganda, reviewed intervention descriptions and implementation in an earlier draft of this manuscript.

Authors' contributions {31b}

HK is the principle investigator; she conceived the study and led the proposal in conjunction with MS. EK, CD, LMB and KFEI contributed to the study design and protocol development for the impact evaluation. CD, TS and AM led development of the process evaluation design and protocol development. SC contributed to the planning of the statistical analysis. EK and CD drafted the initial manuscript. SM substantially revised the manuscript for publication. All authors read and approved the final manuscript.

Funding {4}

The trial is funded through the United Kingdom Foreign, Commonwealth and Development Office (FCDO) under the LSHTM led Programme for Evidence to Inform Disability Action (PENDA). Implementation of the interventions is through a separate project delivered by BRAC Uganda in partnership with Humanity and Inclusion (HI) and the National Union of Women with Disabilities of Uganda (NUWODU), with funding from FCDO under Inclusive Futures, Comic Relief and National Lottery. Hannah Kuper is funded by a National Institute for Health and Care Research (NIHR) Global Research Professorship. Part of Lena Morgon Banks' time and the Open Access fee is covered by the Arts & Humanities Research Council (grant: AH/X009580/1).

Availability of data and materials {29}

Twelve months after the completion of the endline data collection, the data will be made available on LSHTM's Data Compass, along with project documentation and a data user guide. The data will be made available open access, ensuring that no identifiers are included in the data. Explicit consent has been included for making data open access.

Declarations

Ethics approval and consent to participate {24}

The protocol received ethical approval from the Mildmay Uganda Research Ethics Committee (Reference: 0604–2020, Approved: 06/07/2020, Amended: 16/06/2022), London School of Hygiene and Tropical Medicine Research Ethics Committee (Reference: 22619/RR/21198, Approved: 21/09/2020, Amended: 10/05/2023; and for process evaluation under the POInT project, Reference: 28134; Approved: 03/01/2023) and a research permit from the Uganda National Council for Science and Technology (UNCST) (Reference: SS529ES, Received: 22/10/2020). All participants will be required to provide written, informed consent to participate in the study.

Consent for publication {32}
Model consent forms are provided in Supplemental file 1.

Competing interests {28}
The authors declare that they have no competing interests.

Author details
[1]Independent Evaluation and Research Cell (IERC), BRAC International, Kampala, Uganda. [2]Trinity College Dublin, Dublin, Ireland. [3]International Centre for Evidence in Disability, London School of Hygiene & Tropical Medicine, London, UK. [4]Centre for Evaluation, London School of Hygiene & Tropical Medicine, London, UK. [5]Department of Sociology and Anthropology, Makerere University, Kampala, Uganda. [6]BRAC International, Dhaka, Bangladesh. [7]BRAC Institute of Governance and Development, BRAC University, Dhaka, Bangladesh.

Received: 25 August 2023 Accepted: 7 March 2024
Published online: 21 March 2024

References
1. Banerjee A, Duflo E, Goldberg N, Karlan D, Osei R, Parienté W, et al. A multifaceted program causes lasting progress for the very poor: Evidence from six countries. Science. 2015;348(6236):1260799.
2. Hashemi SM, De Montesquiou A. Reaching the poorest: lessons from the graduation model. Focus Note: No.69. CGAP: Washington DC; 2011. Available from: https://www.cgap.org/sites/default/files/CGAP-Focus-Note-Reaching-the-Poorest-Lessons-from-the-Graduation-Model-Mar-2011.pdf.
3. Matin I, Sulaiman M, Rabbani M. Crafting a graduation pathway for the ultra poor: lessons and evidence from a BRAC Programme in Bangladesh. In: Cook S, Kabeer N, editors. Social protection as development policy. London: Routledge India; 2010.
4. Banks LM, Davey C, Shakespeare T, Kuper H. Disability-inclusive responses to COVID-19: Lessons learnt from research on social protection in low- and middle-income countries. World Dev. 2021;137:105178.
5. World Health Organization (WHO). World report on disability 2011. WHO: Geneva; 2011. Available from: https://www.who.int/teams/noncommunicable-diseases/sensory-functions-disability-and-rehabilitation/world-report-on-disability.
6. Banks LM, Kuper H, Polack S. Poverty and disability in low- and middle-income countries: a systematic review. PLoS ONE. 2017;12(12):e0189996-e.
7. Banks LM, Mearkle R, Mactaggart I, Walsham M, Kuper H, Blanchet K. Disability and social protection programmes in low- and middle-income countries: a systematic review. Oxf Dev Stud. 2017;45(3):223–39.
8. Washington Group on Disability Statistics - question sets Washington, USA. Washington Group on Disability Statistics. Available from: https://www.washingtongroup-disability.com/question-sets/.
9. Uganda Bureau of Statistics (UBOS). The National Population and Housing Census 2014 - Main Report. UBOS: Kampala; 2016. Available from: https://www.ubos.org/wp-content/uploads/publications/03_20182014_National_Census_Main_Report.pdf.
10. Banerjee A, Karlan D, Osei R, Thuysbaert B, Udry C. Graduation from ultra poverty in Ghana, 3ie grantee final report. 3ie: New Delhi; 2017. Available from: https://www.3ieimpact.org/sites/default/files/2018-08/3ie%20Grantee%20Final%20Report%20OW2.206.pdf.
11. Sulaiman M, Matin I. Targeting effectiveness of CFPR/TUP in scale-up environment. CFPR/TUP Working Paper Series: No.8. BRAC: Gazipur; 2006. Available from: https://bigd.bracu.ac.bd/wp-content/uploads/2020/03/Targeting-Effectiveness-of-CFPR-TUP-in-Scale-up-Environment.pdf.
12. World Health Organization (WHO). Model Disability Survey (MDS): Survey manual. WHO: Geneva; 2017. Available from: https://www.who.int/publications/i/item/9789241512862.
13. Davey C, Juden M, Allan E, Prost A, Humphreys M, Jacobs A, et al. POInT research design paper. CEDIL: London and Oxford; 2022. Available from: https://cedilprogramme.org/download/8810/.
14. Kuper H, Saran A, White H. Rapid Evidence Assessment (REA) of what works to improve educational outcomes for people with disabilities in low-and middle-income countries. Campbell Collaboration and International Centre for Evidence and Disability: New Delhi and London; 2018. Available from: https://assets.publishing.service.gov.uk/media/5b8ea83040f0b67d9a6fe669/Education_Rapid_Review_full_report.pdf.
15. Roland M, Torgerson DJ. Understanding controlled trials: what are pragmatic trials? BMJ. 1998;316(7127):285.
16. Bates MA, Glennerster R. The generalizability puzzle. Stanf Soc Innov Rev. 2017;15(3):50–4.

Publisher's Note
Springer Nature remains neutral with regard to jurisdictional claims in published maps and institutional affiliations.
